# Comparative and evolutionary analysis of *RIP* kinases in immune responses

**DOI:** 10.3389/fgene.2022.796291

**Published:** 2022-10-03

**Authors:** Shangge Lv, Yu Jiang, Yuzheng Li, Ruilin Huang, Lingyu Peng, Zhaoyin Ma, Nan Lu, Xiaoying Lin, Jie Yan

**Affiliations:** ^1^ Department of Diagnostics, Medical Integration and Practice Center, Cheeloo College of Medicine, Shandong University, Jinan, China; ^2^ Division of Epidemiology, Biostatistics, and Environmental Health, School of Public Health. University of Memphis, Memphis, TN, United States; ^3^ College of Land Science and Technology, China Agricultural University, Beijing, China

**Keywords:** RIP kinases, evolution, comparative analysis, immune responses, ripk3

## Abstract

The group of receptor-interacting protein (RIP) kinases has seven members (*RIPK1–7*), with one homologous kinase domain but distinct non-kinase regions. Although *RIPK1–3* have emerged as key modulators of inflammation and cell death, few studies have connected *RIPK4–7* to immune responses. The divergence in domain structures and paralogue information in the Ensembl database have raised question about the phylogeny of *RIPK1–7*. In this study, phylogenetic trees of RIPK1–7 and paralogues constructed using full-length amino acid sequences or Kinase domain demonstrate that RIPK6 and RIPK7 are distinct from RIPK1–5 and paralogues shown in the Ensembl database are inaccurate. Comparative and evolutionary analyses were subsequently performed to gain new clues about the potential functions of *RIPK3–7*. *RIPK3* gene loss in birds and animals that undergo torpor, a common physiological phenomenon in cold environments, implies that *RIPK3* may be involved in ischemia-reperfusion injury and/or high metabolic rate. The negligible expression of *RIPK4* and *RIPK5* in immune cells is likely responsible for the lack of studies on the direct role of these members in immunity; *RIPK6* and *RIPK7* are conserved among plants, invertebrates and vertebrates, and dominantly expressed in innate immune cells, indicating their roles in innate immunity. Overall, our results provide insights into the multifaceted and conserved biochemical functions of *RIP* kinases.

## Introduction

The innate immune system, as the first line of host defense against infection, is equipped with innate sensors that can effectively clear pathogens by recognizing molecular structures known as pathogen-associated molecular patterns (PAMPs) or danger-associated molecular patterns (DAMPs) (Silke et al., 2015). These innate sensors, including Toll-like receptors, NOD-like receptors and RIG-I-like receptors, are engaged by relevant PAMPs from bacteria, fungi or viruses. This can trigger various intracellular signaling cascades to activate transcription factors, such as nuclear factor-kappa B (NF-κB), activator protein-1 (AP-1), and interferon regulatory factors (IRFs) to induce the production of inflammatory cytokines, chemokines and interferons, as well as activate cell death to eliminate pathogen-infected or damaged cells (Chen et al., 2009; Loo and Gale, 2011; O’Neill et al., 2013; Bhat and Fitzgerald, 2014). Cell death comes in many different forms: apoptosis, which is widely considered silent for inflammation; accidental necrosis and programmed necrosis (such as necroptosis and pyroptosis), which are considered highly inflammatory (Humphries et al., 2015; Li et al., 2021). Therefore, cell death can be closely integrated with inflammation to maintain immune homeostasis (Wallach et al., 2014). Receptor-interacting protein (*RIP*) kinases have emerged as key molecules in the regulation of inflammation and cell death pathways (He and Wang, 2018).

**FIGURE 1:**
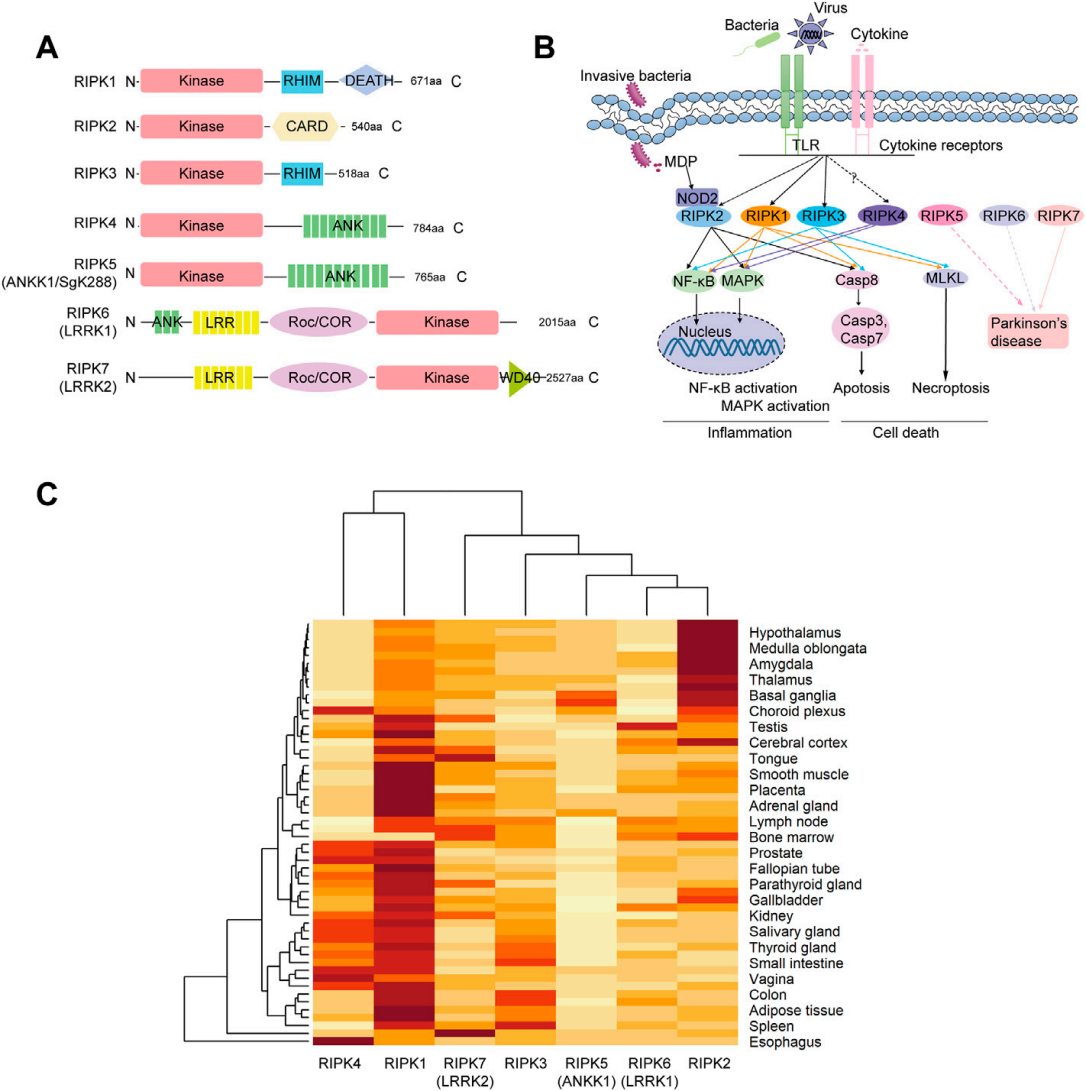
Structure organization, signaling pathways and tissue expression of human *RIP* kinases **(A)** Domain architecture. *RIPK1* ***–*** *7* contains a serine-threonine kinase domain (Kinase). RHIM, RIP homotypic interaction motif; CARD, caspase-activation and recruitment domain; ANK, ankyrin repeats; LRR, leucine-rich repeats; Roc/COR, Ras of complex proteins/C-terminal Roc; WD40, WD40 repeats **(B)** Diagram of signaling networks shown on the KEGG pathway website and in previous studies. NF-kB, nuclear factor-kappa B; MAPK, mitogen-activated protein kinase; NOD, nucleotide-binding oligomerization domain. **(C)** Hierarchical clustering of the expression profiles of *RIPK1* ***–*** *7* in different tissues derived from the consensus data sets of HPA, GTEx and FANTOM5 in the Human Protein Atlas. Expression values are normalized as transcripts per million levels. Hierarchical clustering was conducted on both the row variable (tissue) and the column variable (gene) by heatmap function in R.4.1.0.

*RIP* kinases have seven members (*RIPK1–7*) that share a conserved serine-threonine kinase domain but have distinct non-kinase functional features (Figure 1A) (He and Wang, 2018). *RIPK1*, the first identified member of the *RIP* kinases, contains the following functional features: an N-terminal Kinase domain, which can mediate autophosphorylation to promote its own activation; a C-terminal DEATH domain, which can bind to several death receptors, such as tumor necrosis factor receptor 1 (TNF-R1); and an intermediate RIP homotypic interaction motif (RHIM), which can recruit RIPK3 through activation of IRFs (Ofengeim and Yuan, 2013; Meng et al., 2018; Xu et al., 2018). *RIPK1* mutations are associated with arthritis, inflammatory bowel disease (IBD), recurrent fevers and lymphadenopathy (Cuchet-Lourenço et al., 2018; Li et al., 2019). *RIPK2*, the second characterized member, bears an N-terminal Kinase and a C-terminal caspase-activation and recruitment domain (CARD), through which it can interact with the CARDs of the intracellular peptidoglycan sensors NOD1 and NOD2, resulting in the activation of NF-κB and mitogen-activated protein kinase (MAPK; 15). Recent experimental and clinical studies have provided evidences that *RIPK2* is highly associated with leprosy, osteoarthritis and IBD (Zhang et al., 2009; Thiébaut et al., 2011; Jurynec et al., 2018). *RIPK3*, the third described *RIP* kinase, is composed of N-terminal Kinase and C-terminal RHIM domain, through which RIPK3 can interact with RIPK1 (Sun et al., 1999).

However, few studies have linked immune responses to functions of *RIPK4–7*. *RIPK4* was first identified as protein kinase C (PKC)δ-interacting protein kinase by yeast two-hybrid system (Bähr et al., 2000). RIPK4 has an N-terminal Kinase domain and a C-terminal ankyrin repeats domain (ANK). Overexpression of RIPK4 can induce NF-κB activation and Jun N-terminal kinase (JNK) signaling (Meylan et al., 2002). RIPK5, also known as ankyrin repeat and kinase domain containing 1 (*ANKK1*) or sugen kinase 288 (*SgK288*), harbors a Kinase domain in N terminus and ANK in C terminus (He and Wang, 2018). The ANKK1 TaqIA polymorphism is the most studied genetic variant related to neuropsychiatric disorders (Koeneke et al., 2020; Ohira et al., 2022). Furthermore, ANKK1 overexpression can affect phases of the cell cycle, especially G1 and M (España-Serrano et al., 2017). *RIPK6* and *RIPK7*, also known as *LRRK1* and *LRRK2*, are characterized by leucine-rich repeats (LRR), a Roc GTPase and COR dimerization motif (Roc/COR), and a C-terminal Kinase domain. RIPK6 harbors an ANK motif in the N terminus, and RIPK7 has a WD40 in the C terminus. The LRR motif may play a role in the recognition of molecular patterns associated with damage, pathogens, or stress (Festjens et al., 2007; Zhang et al., 2010). The binding of GTP to Roc/COR domain can stimulate RIPK6 kinase activity (Korr et al., 2006) and assist *RIPK7* in membrane trafficking via the endo-lysosomal pathway (Shin et al., 2008; Piccoli et al., 2011; Sheehan and Yue, 2019). Although many studies reported that *RIPK7* variants and kinase activity is likely central to the pathogenesis of Parkinson’s disease, the basic functions of *RIPK7* remain poorly understood (Berwick et al., 2019; Seegobin et al., 2020). A RIPK6 variant has also been proposed as a risk factor for the development of familial Parkinson’s disease (Dachsel et al., 2010; Schulte et al., 2014).

In summary, *RIPK1–7* exhibit significant divergence in domain structures and functions. Indeed, the common Kinase domain they share is also present in multiple other kinases annotated in the Ensembl database. Some interesting questions remain: What is the evolutionary relationship among *RIPK1–7*? Are the paralogues shown in the Ensembl database incorrect? Although *RIPK1–3* have been adequately reported in human and mice and characterized with similar functions in pig, chicken, frog, zebrafish, black carp and lamprey, they remain poorly investigated in other vertebrate species (Ishizawa et al., 2006; Chang et al., 2010; Park et al., 2018; Hou et al., 2020; Xie et al., 2020; Liu et al., 2021). Meanwhile, little is known about *RIPK4–7* roles in immune responses. In this study, phylogenetic trees of RIPK1–7 and paralogues constructed using their full length and their kinase domain indicated that RIPK6 and RIPK7 are clearly distinct from RIPK1–5 and that the paralogues in the Ensembl database are inaccurate. An extensive BLAST survey identified the ancient eukaryotic ortholog of *RIP* kinases in the protist taxa Cryptophyta (*Guillardia theta*), which suggests that *RIP* kinases arose prior to the separation of animal, plant and fungal lineages. The negligible expression of *Ripk4* and *Ripk5* (*Ankk1*) in immune cells may explain why there have been no studies of the direct role of *Ripk4* and *Ripk5* in immune responses. Furthermore, the close evolutionary relationships of gene expression in organs between *RIPK1* and *RIPK4*, and the fact that 10 of 12 critical residues are homologous between RIPK1 and RIPK4, suggests that *RIPK4* might play a role on NF-κB or MAPK activation like *RIPK1,* but mainly in non-immune cell types. RIPK6 and RIPK7 with the domain structure of the ANK-LRR-Roc/COR-Kinase axis are highly conserved among plants, invertebrates and vertebrates, and markedly expressed in innate immune cells, suggesting their potential dominant role in innate immune responses.

## Material and methods

### Gene extraction

*RIP* kinases in *Homo sapiens* were derived from NCBI (http://www.ncbi.nlm.nih.gov/sites/entrez) as follows: amino acid sequences of RIPK1 (GenBank accession number: NP_001341859), RIPK2 (NP_003812), RIPK3 (NP_006862), RIPK4 (NP_065690), RIPK5 (NP_848605), RIPK6 (NP_078928), and RIPK7 (NP_940980). Paralogues of RIP kinases were extracted from “Paralogues” in Comparative Genomics in the Ensembl database (https://asia.ensembl.org/index.html). According to the established taxonomic relationships, representative organisms from Mammal (human *H. sapiens* and mouse *Mus musculus*), Ave (chicken *Gallus gallus*), Reptilia (green anole *Anolis carolinensis*), Amphibian (clawed frog *Xenopus tropicalis*), Teleost fish (zebrafish *Danio rerio*), Cyclostomata (lamprey *Lethenteron japonicum*), Cephalochordate (amphioxus *Branchiostoma floridae*), Arthropoda (fruitfly *Drosophila grimshawi*), Nematomorpha (nematode *Caenorhabditis elegans*), Cnidaria (fresh-water polyp *Hydra vulgaris*), Amoebozoa (soil-dwelling amoeba *Dictyostelium discoideum*), Cryptophyta (cryptomonad algae *Guillardia theta*), and Plants (silver myrtle *Rhodamnia argentea*) were selected. *RIP* kinase homologs of these species were retrieved based on the best hits of an extensive BLASTP against NCBI and JGI database (https://genome.jgi.doe.gov/portal/) with human RIPK1–7 as the queries. All returned sequences were reciprocally searched against other genomes to further verify their identities (identity ≥ 30%, E-value ≤ 1e-3).

### Gene expression in organs

Expression patterns of *RIPK1–7* mRNA in various human tissues and organs were obtained from the Human Protein Atlas (HPA; https://www.proteinatlas.org/). Expression was normalized as transcripts per million. A heatmap was created using heatmap function in R.4.1.0. With the heatmap function, hierarchical clustering was conducted on both the row variable (tissue) and the column variable (gene). Both tissue and gene names were reordered based on the results of the hierarchical analysis.

### Immune features analysis

Values for the expression of mouse *Ripk1–7* mRNA normalized by DEseq2 in various immune cells were extracted from Gene Skyline from the Immunological Genome Project (ImmGen; http://rstats.immgen.org/Skyline/skyline.html). Correlated gene of *Ripk1–7* in immune cells were obtained with Gene Constellation (http://rstats.immgen.org/GeneConstellation/index.html). An intersection analysis of correlated genes was performed in EVenn (http://www.ehbio.com/test/venn). In addition, correlated genes were uploaded to the Database for Annotation, Visualization and Integrated Discovery (DAVID, https://david.ncifcrf.gov/) with the settings of selected identifier (“OFFICAL_GENE_SYMBOL”), species (“*Homo sapiens*”), and the functional annotation chart to analyze enriched Kyoto Encyclopedia of Genes and Genomes (KEGG) pathways with *p* < 0.05.

### Sequence and functional motif analysis

Functional domains were identified by SMART (http://smart.emblheidelberg.de), which uses HMMER (biosequence analysis using profile hidden Markov models) together with Pfam (http://pfam.sanger.ac.uk/search) for searching domain homologs by default thresholds. Identify of amino acids of RIP kinases between human and other species were determined by pairwise sequence alignment in EMBL-EBI (https://www.ebi.ac.uk/). Functional motif analyses were calculated by MEME (http://meme-suite.org/tools/meme) with a motif size between 6 and 50 amino acids and a maximum of 25 motifs. Alignment was performed with ClustalW from the UGENE server. Signaling pathways regulated by *RIP* kinase were analyzed by KEGG database (https://www.genome.jp/kegg/pathway.html).

### Evolutionary analysis

Maximum likelihood (ML) phylogenetic trees were constructed using the JTT model by MEGA X software. The reliability of each interior branch was assessed by bootstrapping with 100 replications. The gene gain/loss tree of *RIPK3* was derived from Comparative Genomics in the Ensembl database. To indicate whether neighboring genes surrounding *RIPK1–7* were evolutionarily conserved, we performed synteny analysis of surrounding genes with transcriptional orientations using genomic data from NCBI Map Viewer assemblies (http://www.ncbi.nlm.nih.gov/mapview/) for *H. sapiens*, *X. tropicalis*, *B. floridae*, *C. elegans* and *D. discoideum*.

## Results

### Structure organization, expression patterns and functions of human *RIPK1–7*

The immune system protects the host against infection and tissue injury by initiating various cellular signaling pathways (He and Wang, 2018). According to the KEGG database, when innate sensors recognize stimuli (bacteria, viruses, and cytokines), *RIPK1* can be recruited to the signaling complex to elicit inflammation (*NF-κB* and *MAPK*) pathways as well as cell death (necroptosis and apoptosis). *RIPK2* can be recognized by the intracellular receptor *NOD2* and subsequently induce *NF-κB* activation, *MAPK* activation, and apoptosis (Figure 1B). *RIPK3* is involved in apoptosis, necroptosis, and a “non-canonical” NF-κB activation via facilitating RelB-p50 heterodimer nuclear translation in specific cell types, including bone marrow derived dendritic cells and aortic smooth muscle cells (Moriwaki and Chan, 2017). *RIPK7* is associated with Parkinson’s disease. Unfortunately, there is no information about *RIPK4–6* in the KEGG database. However, Meylan *et al.* (Meylan et al., 2002) found that *RIPK4* overexpression can induce *NF-κB* activation and *JNK* signaling (*MAPK* activation). *RIPK5/ANKK1* polymorphisms are associated with neuropsychiatric disorders, including Parkinson’s disease (Ohira et al., 2022). A RIPK6 variant (p. Arg1261Gln) has also been identified as a candidate for a disease-causing genetic variant of Parkinson’s disease (Schulte et al., 2014).

Furthermore, expression patterns of *RIP* kinases in various tissues were obtained from HPA using a combination of the HPA, GTEx, and FANTOM5 (Functional Annotation of the Mammalian Genome 5) data sets. As shown in Supplementary Figure S1, *RIPK1* and *RIPK2* are widely detected in all tissues. *RIPK3*, *RIPK4*, *RIPK5*, *RIPK6*, and *RIPK7* are detected in many tissues but enhanced in intestine, esophagus, skin/brain, lymphoid tissue, and lung, respectively. Moreover, we used hierarchical analysis to dissect the correlation between expression data and phylogeny. As shown in Figure 1C, the heatmap analysis showed that the phylogeny of *RIPK4* expression is close to that of *RIPK1*, which are distinct from the other five members. Meanwhile, phylogeny of *RIPK6* is close to that of *RIPK2*.

### Associations between human *RIPK1–7* and immune cell signatures

**FIGURE 2:**
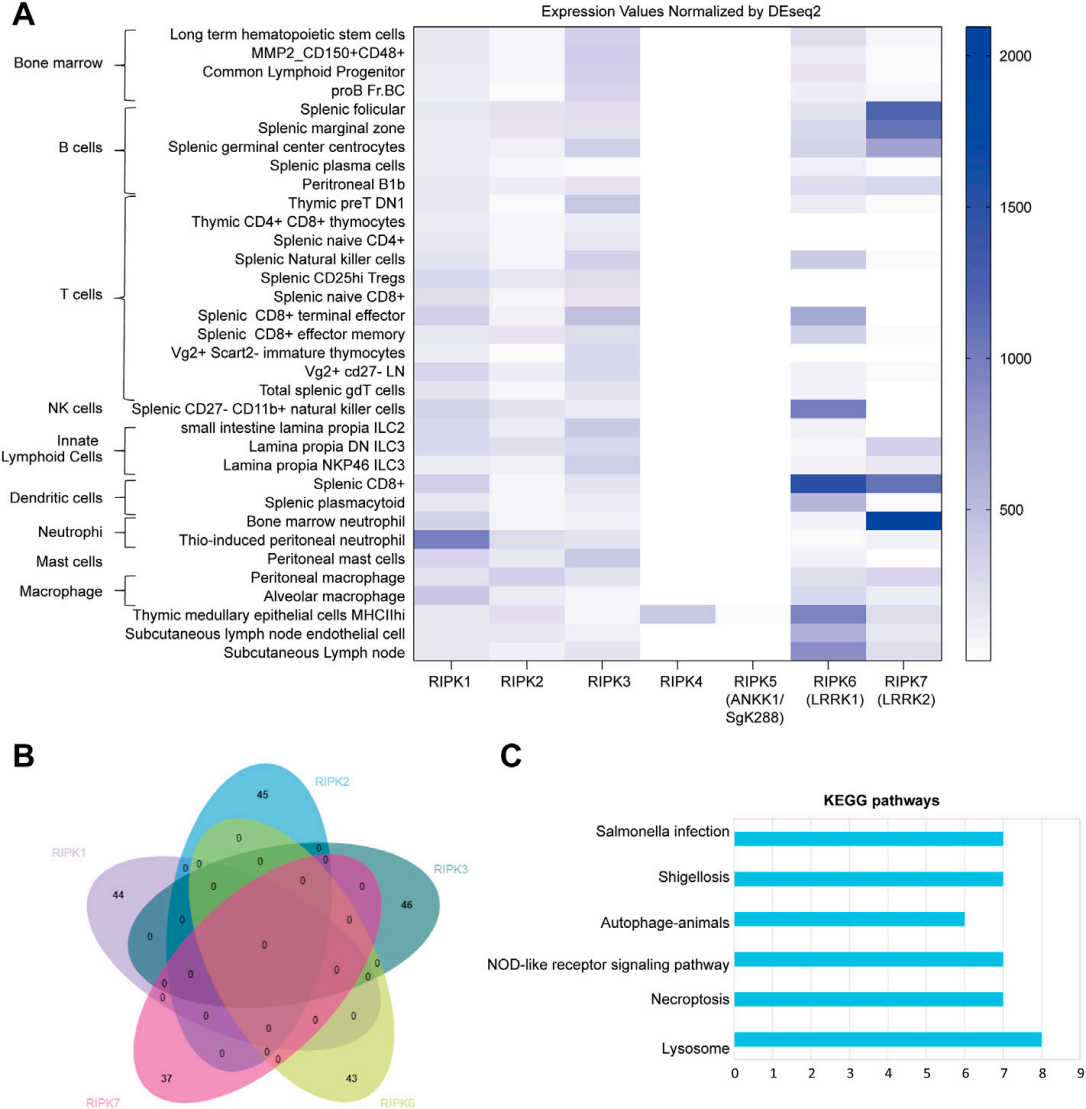
Mouse *Ripk1* ***–*** *7* in immune cells **(A)** Expression pattern of *Ripk1* ***–*** *7* normalized by DESeq2 in immune cells derived from Gene Skyline in ImmGen, including bone marrow, B cells, T cells, natural killer cells, innate lymphoid cells, neutrophils, dendritic cells, macrophages, and mast cells. **(B)** Intersection analysis of genes significantly correlated with *Ripk1* ***–*** *7* in immune cells extracted from Gene Constellation by EVenn. **(C)** KEGG pathway enrichment analysis of the correlated gene with *Ripk1* ***–*** *7*. The *x* axis shows the number of genes clustered in each category. The *y* axis shows the names of the enriched signaling pathways. *p* is set at 0.05.

Currently, very few studies have connected inflammation and cell death with *RIPK4–7*. We therefore analyzed expression profiles of mouse *Ripk1–7* in various mouse immune cells from the ImmGen server, including bone marrow, B cells, T cells, natural killer cells, innate lymphoid cells, neutrophils, dendritic cells, macrophages, and mast cells. As shown in Figure 2A, *Ripk1* is enriched in neutrophils and widely detected in all cells. *Ripk2* and *Ripk3* are distributed in all immune cells. We were surprised to find that *Ripk4* and *Ripk5* are negligible in all immune cells except MHCII^high^ thymic medullary epithelial cells, which indicates an insignificant role of *Ripk4* and *Ripk5* in immune responses. *Ripk6* is dominant in dendritic cells and natural killer cells, expressed at a low level in innate lymphoid cells, and missing in some CD4^+^ and CD8^+^ T cells. *Ripk7* is mainly expressed in neutrophils and B cells and is absent in T cells, natural killer cells, and ILC2 cells, which suggests the predominant role of *Ripk7* in innate immune responses.

To further investigate the potential molecular mechanism of RIP kinase in these immune cells, we extracted genes significantly correlated with *Ripk1–3* and *Ripk6* and *Ripk7* using Gene Constellation in ImmGen; *Ripk4* and *Ripk5* were excluded because of their negligible expression in immune cells. An intersection analysis of the correlated genes in EVenn indicates no common correlated genes between any two RIP kinases (Figure 2B). DAVID showed enriched KEGG pathways for these correlated genes includes pathogen infection (*Salmonella* infection and shigellosis), inflammation (NOD-like receptor signaling pathway, autophagy-animals and lysosome), and cell death (necroptosis) (Figure 2C).

### What is the evolutionary relationship among human RIPK1–7?

**FIGURE 3:**
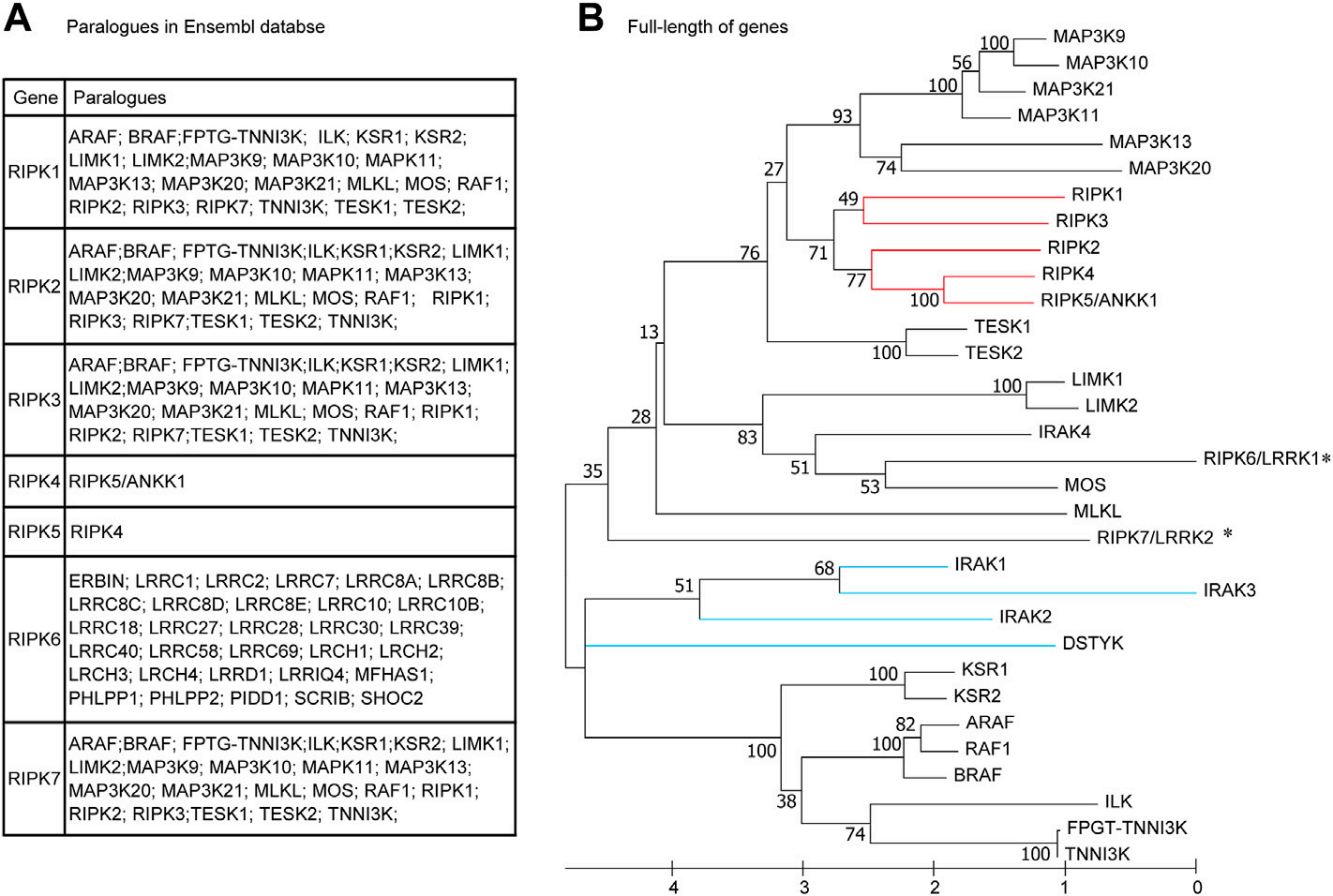
Phylogenetic relationships among human RIPK1–7 and their paralogues **(A)** Paralogues in the Ensembl database. **(B)** The maximum likelihood (ML) tree using the full-length amino acid sequences. The bootstrap percentage derived from 100 replications is shown on the interior branches. GenBank accession numbers of the full-length genes are as follows: IRAK1 (NP_001560), IRAK2 (NP_001561), IRAK3 (NP_009130), IRAK4 (NP_001107654), DSTYK (NP_056190). Other sequence accession numbers refer to Table 1. * indicates human RIPK6 and RIPK7 genes; the red line indicates RIPK1–5; the blue line indicates DSTYK and IRAK1–3.

It is remarkable that RIPK1–7 exhibit salient divergence in their domain structures, expression patterns, and functions, although they share a similar Kinase domain. In the paralogue family in the Ensembl database (Figure 3A), RIPK1–3 and RIPK7 share the same paralogues, whereas RIPK4–6 have completely different paralogues. Furthermore, the paralogues of RIPK1–5 and RIPK7 contain a Kinase domain and other diverse domains/motifs according to SMART, whereas RIPK6 paralogues do not contain a Kinase domain but have an LRR domain instead (Table 1). Moreover, there is some controversy regarding which gene can be represented as *RIPK5*. Dusty protein kinase (*DSTYK*; GenBank accession number: NP_056190) is characterized as *RIPK5* by the majority of public databases (e.g., NCBI and Ensembl) and Zha et al., 2004 (Zha et al., 2004). Kinome analysis by Manning et al., 2002 indicated that DSTYK is closer to IRAKs than to the other RIP genes (Manning et al., 2002). This raises the following questions: What is the evolutionary relationship among human RIPK1–7? Are the paralogues shown in the Ensembl database incorrect?

**TABLE 1:** The overall sequence information of paralogues of human *RIPK1–7* in Ensembl genome database.

Gene	Accession numbers	Length (aa)	Position of kinase domain	Position of DEATH domain	Position of ANK domain	Position of LRR domain	Position of Roc/COR/RBD domain	Position of C1 domain	Position of PDZ domain	Position of other domain
ARAF	NP_001645.1	606	310–568	—	—	—	19–91	99–144	—	—
BRAF	NP_004324.2	766	457–716	—	—	—	155–227	235–280	—	—
FPTG-TNNI3K	NP_001106279.3	936	564–820		167–197; 201–230; 234–263; 267–296; 300–331; 335–366; 370–401; 405–436; 440–469; 482–511	—	—	—	—	—
ILK	NP_001014794.1	452	195–445		33–62; 66–95; 99–128	—	—	—	—	—
KSR1	NP_055053.1	759	476–741		—	—	—	211–254	—	
KSR2	NP_775869.4	950	666–928		—	—	—	411–456	—	**KSR1-SAM:**24–152
LIMK1	NP_002305.1	647	339–604		—	—	—	339–604	176–258	**LIM**:24–75; 83–137
LIMK2	NP_001026971.1	686	310–572		—	—	—	—	140–218	**LIM**:7–42; 50–103; *p* **P1-inhibitor**: 577–686
MAP3K9	NP_149132.2	1118	144–403		—	—	—	—	—	**SH3**: 55–115
MAP3K10	XP_011525283.1	962	98–365		—	—	—	—	—	**SH3**: 19–80
MAP3K11	XP_011525283.1	847	117–376		—	—	—	—	—	**SH3**: 44–104
MAP3K13	NP_001229243.1	966	168–407		—	—	—	—	—	
MAP3K20	NP_057737.2	800	16–259		—	—	—	—	—	**KSR1-SAM:**336–410
MAP3K21	NP_115811.2	1036	124–398		—	—	—	—	—	**SH3**: 41–101
MLKL	NP_689862.1	471	213–466		—	—	—	—	—	—
MOS	NP_005363.1	344	61–337		—	—	—	—	—	—
RAF1	NP_001341618.1	668	369–628		—	—	56–131	139–184	—	—
TNNI3K	NP_057062.1	835	463–719	—	66–96; 100–129; 133–162; 166–195; 199–230; 234–165; 269–300; 304–335; 339–368; 381–410	—	—	—	—	—
TESK1	NP_006276.2	626	57–314	—	—	—	—	—	—	—
TESK2	NP_009101.2	571	60–309	—	—	—	—	—	—	—
ANKK1	NP_848605.1	765	25–285	—	361–390; 394–423; 427–456; 460–489; 493–522; 526–555; 559–588; 592–621; 625–654; 658–687; 691–720	—	—	—	—	—
NRBP1	NP_848605.1	535	81–324	—	—	—	—	—	—	—
NRBP2	NP_848659.2	501	55–306	—	—	—	—	—	—	—
WNK1	NP_061852.3	2382	221–479	—	—	—	—	—	—	—
WNK2	NP_001269323.1	2297	195–451	—	—	—	474–537	—	—	—
WNK3	NP_065973.2	1800	147–405	—	—	—	426–489	—	—	—
WNK4	NP_115763.2	1243	174–430	—	—	—	—	—	—	**OSR1-C:**453–515
ERBIN	NP_001240626.1	1412	—	—	—	48–68; 91–114; 137–159; 160–182; 183–205; 206–228; 229–252; 253–274; 275–298; 321–344; 345–366; 367–389	—	—	—	—
LRRC1	NP_060684.4	524	—	—	—	35–57; 58–80; 83–104; 104–123; 127–146; 150–172; 173–195; 196–218; 219–241; 242–264; 265–288; 288–307; 311–334; 336–357; 358–380	—	—	—	—
LRRC2	NP_078788.2	371	—	—	—	143–165; 166–189; 236–258; 259–282	—	—	—	—
LRRC7	NP_001317564.1	1495	—	—	—	53–73; 96–108; 142–164; 165–187; 188–210; 211–233; 234–257; 258–279; 280–303; 326–349; 372–394	—	—	—	
LRRC8A	NP_001120716.1	810	—	—	—	590–613; 614–636; 638–660; 661–684; 685–706; 707–730; 731–751; 753–776	—	—	—	Pannexin_like: 1–340
LRRC8B	NP_001127948.1	803	—	—	—	509–536; 584–607; 609–630; 632–654; 655–678; 679–700; 701–724; 747–770	—	—	—	Pannexin_like: 1–334
LRRC8C	NP_115646.3	803	—	—	—	588–611; 613–635; 636–658; 659–682; 684–703; 705–728; 751–774	—	—	—	Pannexin_like: 1–338
LRRC8D	NP_001127951.1	858	—	—	—	657–680; 682–704; 705–728; 729–750; 751–774; 775–796; 797–820	—	—	—	Pannexin_like: 1–384
LRRC8E	NP_001255213.1	796	—	—	—	604–628; 629–651; 652–675; 676–697; 698–721; 722–743; 744–767	—	—	—	Pannexin_like: 1–331
LRRC10	NP_963844.2	277	—	—	—	51–73; 74–97; 120–143; 166–189	—	—	—	—
LRRC10B	NP_001138549.1	292	—	—	—	43–65; 66–87; 89–111; 135–156; 158–181	—	—	—	—
LRRC18	NP_001006940.3	261	—	—	—	49–71; 72–95; 120–142; 143–166	—	—	—	—
LRCC27	NP_001137229.1	530	—	—	—	66–89; 90–113; 114–136	—	—	—	—
LRCC28	NP_001308604.1	367	—	—	—	40–63; 64–86; 87–109; 110–132; 133–156; 179–202	—	—	—	—
LRCC30	NP_001099051.1	301	—	—	—	70–92; 93–115; 116–139; 140–161; 162–185; 208–230; 231–254	—	—	—	—
LRCC39	NP_001243314.1	339	—	—	—	105–127; 128–151; 175–197; 198–220; 221–243; 244–267	—	—	—	—
LRCC40	NP_060238.3	602	—	—	—	81–100; 104–126; 127–149; 150–172; 173–195; 196–218; 219–241; 242–264; 288–310; 311–334; 335–356; 471–493; 494–517; 541–564	—	—	—	—
LRCC58	NP_001093148.1	371	—	—	—	44–66; 67–90; 119–141; 142–164; 165–187; 188–210; 211–234	—	—	—	—
LRCC69	NP_001123362.1	347	—	—	—	36–58; 59–81; 82–105; 106–128; 129–150; 152–174; 175–198; 200–221	—	—	—	
LRCH1	NP_001157683.2	763	—	—	—	119–141; 142–165; 187–209; 210–233; 255–278	—	—	—	**CH:** 613–722
LRCH2	NP_065922.3	765	—	—	—	133–155; 156–179; 181–201; 224–247; 269–292	—	—	—	
LRCH3	NP_001350816.1	803	—	—	—	104–126; 127–150; 172–194; 195–218; 240–263	—	—	—	**CH:** 658–762
LRCH4	NP_002310.2	683	—	—	—	90–112; 158–180; 226–249	—	—	—	**CH:** 540–645
LRRD1	NP_001155000.1	860	—	—	—	187–209; 256–278; 279–301; 302–324; 371–394; 395–416; 417–440; 486–509; 532–555; 650–671; 673–695; 696–719; 721–742	—	—	—	—
LRRIQ4	NP_001073929.1	560	—	—	—	47–69; 70–92; 93–116; 117–140; 141–164; 187–209; 210–233; 234–255; 256–279; 302–324; 325–347; 348–371; 397–419; 420–443; 444–466	—	—	—	**IQ:503–525**
MFHAS1	NP_004216.2	1052	—	—	—	62–85; 86–109; 110–129; 134–156; 180–202; 203–225; 226–248; 272–294; 295–317; 318–338; 341–364	411–541	—	—	—
PHLPP1	NP_919431.2	1717	—	—	—	692–713; 713–732; 736–758; 759–781; 782–805; 830–853; 893–912; 916–939; 939–958; 963–984; 985–1004; 1035–1054; 1059–1082	—	—	—	**PH:**537–638;*p* **P2Cc**:1165–1420
PHLPP2	NP_055835.2	1323	—	—	—	298–317; 321–343; 344–366; 367–386; 459–482; 501–520; 524–543; 547–566; 572–592; 593–612; 619–644; 643–662; 667–690; 690–709; 712–736	—	—	—	*p* **P2Cc:**775–1031
PIDD1	NP_665893.2	910	—	778–873	—	124–146; 147–169; 170–192; 193–215; 216–238; 239–261; 262–285	—	—	—	**ZU5:**323–417; 457–545;**Peptidase-S68**:421–453
SCRIB	NP_874365.3	1655	—	—	—	58–80; 81–104; 127–149; 150–172; 173–195; 219–241; 242–265; 335–356; 357–380	—	—	736–815; 870–950; 1012–1093; 1109–1192	—
SHOC2	NP_001311265.1	582	—	—	—	122–144; 145–167; 168–190; 191–213; 214–235; 237–260; 283–306; 307–329; 330–353; 354–377; 401–423; 424–446; 447–469; 470–492; 493–514; 516–540	—	—	—	—

To address these questions, we used MEGA X to construct an ML phylogenetic tree using the full-length amino acid sequences from RIPK1–7, twenty paralogues with a Kinase domain in the Ensembl database, DSTYK, and four IRAK genes (IRAK1–4). As shown in Figure 3B, RIPK1–4 and RIPK5/ANKK1 cluster together, whereas RIPK6 and RIPK7 branch with other genes (e.g., MOS and MLKL), implying RIPK6 and RIPK7 are distantly related to RIPK1–5. Meanwhile, DSTYK branches with IRAK1–3 and other “paralogues” of RIPK1–3 (e.g., KSR1–2, ARAF, and BRAF), suggesting DSTYK is clearly distinct from RIPK1–7 and the paralogue information in the Ensembl database is inaccurate.

**FIGURE 4:**
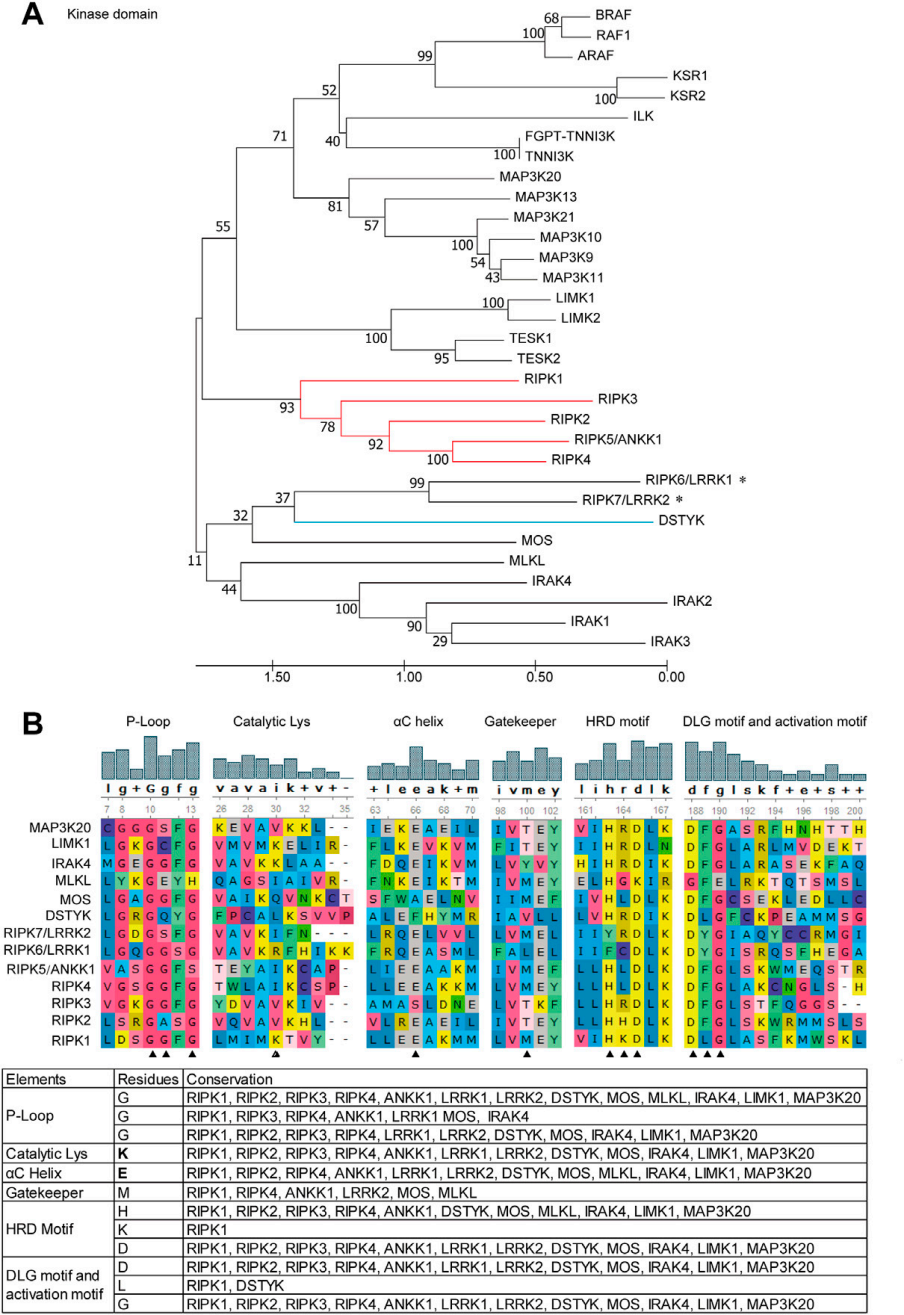
Phylogenetic tree and critical residues of Kinase domains from RIPK1–7 and their paralogues **(A)** ML tree. Branch support values represent a percentage of 100 bootstrap replicates. **(B)** Alignments of the critical catalytic elements by UGENE using ClustalW analysis. Sequence accession numbers used in this analysis refer to Table 1. * indicates human RIPK6 and RIPK7 genes; the red line indicates RIPK1–5; the blue line indicates DSTYK and IRAK1–3; ▲ indicates critical residues.

Furthermore, we dissected the evolutionary relationship of homologous regions, Kinase domain across RIPK1–7 and “paralogues”, using a phylogenetic tree and sequence alignment (Figure 4). Similarly, the ML tree of the Kinase domain exhibits RIPK1–5 clusters together and RIPK6 and RIPK7 with DSTYK in a separate clade (Figure 4A). Moreover, Cuny et al., 2021 identified a total of 12 amino acids in RIPK1 to be critical for the function of Kinase domain, such as canonical catalytic elements including P-loops, catalytic Lys, αC helix, Gatekeeper, HRD motif and DLG motif, and activation loop (Cuny and Degterev, 2021). The sequence alignment of Kinase domain by Clustal-W reveals that the number of conserved critical residues is as follows: RIPK4 and MOS have 10; RIPK5/ANKK1, DSTYK and IRAK4 exhibit 9; RIPK2, RIPK3, RIPK6, RIPK7, MAP3K12 and LIMK1 possess 8; MLKL consists 4 (Figure 4B). Because of possible mismatches, Lys (K) and Glu (E) next to the aligned residues in the alignment were also counted. Importantly, both Lys (K) in the catalytic Lys motif and Glu (E) in the αC helix, as the key catalytic site in the Kinase domain, are detected in RIPK1–7 except RIPK3.

### Evolutionary analysis of RIPK1–7 across different species

In general, an evolutionary framework can provide more insights into multifaceted and conserved functions of genes. In particular, differences in *RIPK1–7* between vertebrates have been poorly characterized, and their evolutionary origins have not been investigated in detail. To define the origin of *RIPK1–7*, we performed an extensive BLASTP search in NCBI and the JGI database using amino acids of human RIPK1–7 to identify homologs in representative organisms (Tables 2, 3). The homologs of RIPK1–7 are not identified in the bacterial genomes examined, which rules out a prokaryotic origin. It is also possible that the current phylogenetic signal is inadequate to identify ancient orthologs. The ancient eukaryotic homologs of RIPK1–7 can be traced back to protist taxa Cryptophyta (*G. theta*) that arose almost two billion years ago (Macqueen and Johnston, 2009; Califice et al., 2012). This places the occurrence of RIPK1–7 ahead of the split of animal, plant, and fungal lineages. Although there are no homologs in fungal genomes, the presence of a homolog of RIP kinases is identified in plants (*R. argentea*). The number of RIPK1–7 homologs varies in different eukaryotic species: seven in human, mouse, and frog (*RIPK1–7*); 6 in chicken with *RIPK3* loss; 6 in sea lamprey (*RIPK1*, *RIPK3*, *RIPK6*, *RIPK7*, two *RIPK2*); 8 in green anole and zebrafish (*RIPK1–7* with two *RIPK2*); 19 in amphioxus; one in fruitfly; one in nematode; four in fresh-water polyp; one in soil-dwelling amoeba, two in cryptomonad algae; and one in plant silver myrtle.

**TABLE 2:** The RIPK1–7 homologs from representative animals in vertebrates.

Gene	Species	Common Name	Accession numbers	Identities to human RIPK1	Length (aa)	Position of Kinase	Position of RHIM	Position of DEATH
RIPK1	*H. sapiens*	Human	NP_001341859.1		671	17-285	504-549	573-669
	*M. musculus*	Mouse	NP_001346926.1	69.80%	656	18-286	480-538	558-654
	*G. gallus*	Chicken	NP_989733.2	48.60%	658	13-281	487-536	560-656
	*A. carolinensis*	Green anole	XP_003224434.1	48.50%	699	13-279	520-574	595-691
	*X. tropicalis*	Frog	NP_001072503.1	42.90%	669	17-288	474-542	564-661
	*D. rerio*	zebrafish	NP_001036815.1	39.00%	661	15-284	480-534	558-654
	*P. marinus*	Sea lamprey	XP_032813622.1	31.90%	744	14-293		651-743

Gene	Species	Common Name	Accession numbers	Identities to human RIPK2	Length (aa)	Position of Kinase	Position of CARD	Position of DEATH
RIPK2	*H. sapiens*	Human	NP_003812.1		540	18-289	435-526	
	*M. musculus*	Mouse	NP_620402.1	84.30%	539	18-290	434-522	
	*G. gallus*	Chicken	NP_001026114.1	63.70%	574	27-295	479-563	
	*A. carolinensis*	Green anole	XP_008106622.1	64.40%	560	38-307	464-548	
			XP_008112157.1	35.00%	392	1-215		
	*X. tropicalis*	Frog	XP_002939201.2	52.80%	553	47-320	462-553	
	*D. rerio*	zebrafish	NP_919392.2	51.30%	584	28-300	470-556	
			XP_005166455.1	27.20%	513	21-264	424-503	
	*P. marinus*	Sea lamprey	XP_032813586.1	51.00%	664	18-287	573-660	
			XP_032816536.1	34.50%	545	26-378		452-541

Gene	Species	Common Name	Accession numbers	Identities to human RIPK3	Length (aa)	Position of Kinase	Position of RHIM
RIPK3	*H. sapiens*	Human	NP_006862.2		518	21-283	417-468
	*M. musculus*	Mouse	NP_064339.2	60.40%	486	22-288	408-458
	*G. gallus*	Chicken	N				
	*A. carolinensis*	Green anole	XP_003223896.2	36.70%	488	16-290	434-478
	*X. tropicalis*	Frog	XP_002934332.3	36.30%	514	13-276	401-439
	*D. rerio*	zebrafish	XP_001343827.1	38.50%	433	19-287	341-403
	*P. marinus*	Sea lamprey	XP_032816533.1	38.00%	634	81-347	

Gene	Species	Common Name	Accession numbers	Identities to human RIPK4	Length (aa)	Position of Kinase	Position of ANK
RIPK4	*H. sapiens*	Human	NP_065690.2		784	22-283	437-466; 470-499; 503-532; 536-565; 569-599; 603-632; 636-665; 669-698; 702-732; 734-763
	*M. musculus*	Mouse	NP_076152.2	90.50%	786	23-283	439-468; 472-501; 505-534; 538-567; 571-601; 605-634; 638-667; 671-700; 704-734; 736-765
	*G. gallus*	Chicken	XP_004934622.2	77.20%	789	23-283	436-485; 489-498; 502-531; 535-564; 568-598; 602-631; 635-664; 668-697; 701-729; 733-762
	*A. carolinensis*	Green anole	XP_003219008.1	76.10%	788	22-283	434-463; 467-496; 500-529; 533-562; 566-596; 600-629; 633-662; 666-695`; 699-727; 731-760
	*X. tropicalis*	Frog	XP_002941332.1	71.40%	717	24-283	438-467; 471-500; 504-533; 537-566; 570-600; 604-633; 637-666; 670-699
	*D. rerio*	zebrafish	NP_998243.1	62.10%	820	23-283	433-462; 466-496; 500-529;533-562; 566-596; 600-629; 633-662; 666-695; 699-728;733-762
	*P. marinus*	Sea lamprey	N				

Gene	Species	Common Name	Accession numbers	Identities to human RIPK5	Length (aa)	Position of Kinase	Position of ANK
RIPK5 (ANKK1)	*H. sapiens*	Human	NP_848605.1		765	25-285	361-390; 394-423; 427-456; 460-489; 493-522; 526-555; 559-588; 592-621; 625-654; 658-687; 691-720
	*M. musculus*	Mouse	NP_001363880.1	79.00%	746	35-297	370-399; 403-432; 436-465; 469-498; 502-531; 535-564; 568-597; 601-630; 634-663; 667-696; 700-729
	*G. gallus*	Chicken	XP_003642663.2	61.50%	830	70-332	414-443; 447-476; 480-509; 513-542; 546-575; 579-608; 612-641; 645-674; 678-707; 711-740; 744-773; 777-806
	*A. carolinensis*	Green anole	XP_008123542.1	38.90%	379 (partial)	16-290	
	*X. tropicalis*	Frog	XP_002937872.1	53.20%	766	30-289	358-387; 391-420; 424-453; 457-486; 490-519; 523-552; 556-585; 589-618; 622-651; 655-684; 688-717; 721-749; 765-794; 798-827
	*D. rerio*	zebrafish	NP_001124137.1	41.90%	733	30-282	347-376; 380-409; 413-442; 446-475; 480-509; 513-542; 546-575; 579-608; 612-641; 645-674
	*P. marinus*	Sea lamprey	N				

Gene	Species	Common Name	Accession numbers	Identities to human RIPK6	Length (aa)	Position of ANK	Position of LRR	Position of Roc/COR	Position of Kinase
RIPK6 (LRRK1)	*H. sapiens*	Human	NP_078928.3		2015	119-148; 152-182; 193-223	278-300; 301-324; 328-351; 379-401; 403-427; 472-493; 548-569; 570-594	623-1046	1243-1520
	*M. musculus*	Mouse	NP_666303.3	88.80%	2014	86-116; 119-148; 162-182; 193-223	278-300; 301-325; 328-351; 379-401; 403-427; 472-493; 548-569; 570-594	625-1046	1244-1520
	*G. gallus*	Chicken	NP_001376322	77.6%	1998	76-105; 109-138; 142-172; 182-212	267-286; 291-310; 318-337; 369-388; 392-412; 441-462; 462-481; 537-556; 560-581;	630-1036	1231-1509
	*A. carolinensis*	Green anole	XP_008116735.1	73.50%	2000	76-106; 109-139; 142-172; 183-212	268-290;291-313; 318-341; 369-392; 393-417; 462-483; 536-558; 560-584	630-1034	1233-1510
	*X. tropicalis*	Frog	XP_012815031.2	67.50%	2003	110-139; 143-173; 184-213	270-289; 294-313; 321-340; 371-390; 395-414; 464-488; 539-558; 562-582	632-1038	1233-1512
	*D. rerio*	zebrafish	XP_021333791.1	56.90%	2007	107-137; 140-169; 173-201; 214-243	298-317; 322-346; 349-368; 400-423; 424-443; 448-464; 472-489; 493-512; 566-585; 613-636	659-1067	1267-1498
	*P. marinus*	Sea lamprey	XP_032806825.1	41.80%	1825		59-78; 83-102; 132-151; 161-184; 185-204; 231-250; 254-273; 339-358; 362-382	434-883	1080-1361

Gene	Species	Common Name	Accession numbers	Identities to human RIPK7	Length (aa)	Position of ANK	Position of LRR	Position of Roc/COR	Position of Kinase	Position of WD40
RIPK7 (LRRK2)	*H. sapiens*	Human	NP_940980.4		2527		1010-1033; 1034-1057; 1082-1105; 1128-1151; 1195-1219; 1244-1266; 1267-1291	1319-1740	1882-2132	2231-2276
	*M. musculus*	Mouse	NP_080006.3	86.60%	2527	708-737; 770-800	1010-1033; 1034-1057; 1082-1105; 1128-1151; 1195-1219; 1244-1266; 1267-1291	1336-1740	1882-2132	2231-2274; 2401-2438
	*G. gallus*	Chicken	NP_001274122.2	72.60%	2557	736-765; 798-828	1039-1062; 1063-1082;1111-1130; 1135-1154; 1157-1176; 1181-1200; 1201-1220; 1224-1243; 1274-1291; 1296-1316	1364-1769	1904-2161	
	*A. carolinensis*	Green anole	XP_008109800.1	72.10%	2540	719-748; 781-811	1022-1045; 1046-1069; 1094-1116; 1140-1163; 1183-1206; 1207-1231; 1256-1279	1332-1752	1892-2144	
	*X. tropicalis*	Frog	XP_002932250.3	62.40%	2514	406-435; 705-734; 737-768	1002-1024; 1026-1045; 1074-1093; 1120-1143; 1187-1206; 1237-1255; 1259-1279	1329-1732	1867-2124	
	*D. rerio*	zebrafish	NP_001188385.2	47.70%	2556	735-764; 767-798	1026-1045; 1050-1073; 1098-1117; 1122-1141; 1144-1163; 1188-1207; 1211-1235; 1261-1279; 1283-1303	1351-1750	1889-2146	2215-2248; 2251-2296; 2359-2399
	*P. marinus*	Sea lamprey	XP_032810325.1	38.30%	2533	736-765; 768-800	984-1004; 1008-1027; 1056-1078; 1080-1100; 1102-1122; 1146-1165; 1169-1193; 1219-1237; 1241-1264	1307-1710	1864-2120	

“N” means not found.

**TABLE 3:** The orthologue of RIP kinases from representative animals in invertebrates.

Phylum	Species	Common name	Gene	Accession numbers	Length (aa)	Position of ANK	Position of LRR	Position of Roc/COR	Position of kinase	Position of DEATH	Position of WD40	Position of other domain
Choanozoa	*B. floridae*	Amphioxus	LOC118408564	XP_035665266.1	254	—	—	—	1–225	—	—	—
			LOC118428708	XP_035694742.1	458	—	—	—	156–415	—	—	**Zalpha**: 1–63
			LOC118408549	XP_035665251.1	344	—	—	—	58–319	—	—	—
			LOC118417619	XP_035679122.1	539	—	—	—	17–280	448–539	—	—
			LOC118418091	XP_035679812.1	705	—	—	—	20–284	614–705	—	—
			LOC118408554	XP_035665256.1	385	—	—	—	88–356	1–41	—	—
			LOC118406448	XP_035662392.1	1250	—	55–74; 78–101; 150–169; 173–196; 245–264; 268–287; 291–315	—	956–1222	—	—	**ZU5**: 448–546
			LOC118408279	XP_035664857.1	678	—	95–111; 115–134; 138–158; 161–180; 184–203; 256–275; 279–298; 302–321		392–656			
			LOC118408570	XP_035665271.1	593	—	14–37; 63–86; 109–132		307–568	164–259	—	—
			LOC118408558	XP_035665259.1	918		52–68; 72–91; 95–114; 118–137; 141–160; 164–183; 187–206; 210–233; 258–277; 281–300; 304–323		633–894	495–586	—	—
			LOC118408552	XP_035665254.1	801		38–54; 58–77; 81–100; 104–123; 127–146; 150–169; 173–192; 196–215; 219–238; 242–265; 291–310; 314–336; 337–356		535–796	392–487		
			LOC118408550	XP_035665252.1	1210	—	38–54; 58–77; 81–100; 104–123; 127–146; 150–169; 166–188; 189–208; 212–231; 235–254; 304–323; 327–346; 350–369; 375–398; 873–889; 893–912; 916–935; 939–958; 962–981; 985–1004; 1008–1027; 1031–1050; 1054–1073; 1100–1119; 1123–1142; 1146–1165; 1171–1192		548–809	405–500	—	—
			LOC118408562	XP_035665264.1	758	—	37–53; 57–76; 80–99; 103–125; 126–145; 152–171; 224–243; 247–269; 270–289	—	468–729	325–420	—	—
			LOC118408547	XP_035665248.1	1373	—	38–54; 58–77; 81–100; 104–123; 127–146; 150–173; 186–205; 209–228; 232–251; 255–274; 278–297; 301–324; 350–369; 386–415; 894–913	—	592–653; 1083–1344	451–546; 949–1044	—	—
			LOC118408560	XP_035665262.1	891	—	38–54; 58–77; 82–104; 104–123; 127–146; 150–169; 173–192; 196–215; 219–238; 242–261; 265–284; 288–311; 357–376; 403–422		601–862	458–553	—	—
			LOC118408566	XP_035665268.1	845	—	89–112; 115–131; 135–154; 158–177; 181–204; 216–235; 239–258; 262–285; 311–330; 334–353; 357–376	—	555–816	412–507	—	**SH3**: 7–62
			LOC118409909	XP_035667179.1	2143	270–299; 302–331; 335–364; 367–397; 399–428	510–529; 563–582; 604–623; 651–667; 696–715; 720–741; 792–811	877–1308	1494–1810	—	—	—
			LOC118416238	XP_035677216.1	2597	678–707; 710–740	1187–1210; 1211–1234; 1258–1277; 1282–1305; 1306–1325; 1330–1349; 1354–1382	1432–1835	1972–2221	—	—	**ARM**: 130–172; 173–216; 459–501
			LOC118422684	XP_035686272.1	2680	43–72; 79–108; 112–141; 180–211; 213–242; 248–277; 296–325; 355–384; 409–439	563–582; 587–606; 614–633; 637–656; 660–679; 788–811; 812–930; 987–1006	1113–1495	1972–2266	—	—	—
Arthropoda	*D. grimshawi*	Hawaiian fruitfly		XP_032596557.1	2469	81–112; 114–143; 148–178; 310–339; 359–389	495–518; 518–537; 544–563; 567–586; 590–609; 684–703; 709–731; 731–750; 805–828; 852–873; 875–899	992–1423	1748–2045			
Nematomorpha	*C. elegans*	Worm	Irk-1	NP_492839.4	2393	56–86; 90–119; 197–226; 230–259; 264–293; 317–347; 361–390; 407–437	530–553; 579–601; 602–625; 626–650; 740–763; 854–878; 881–904	977–1428	1738–1986	—	—	—
Cnidaria	*H. vulgaris*	Fresh-water polyp	—	XP_012555867.1	2064	54–84; 88–117; 133–164; 185–214; 225–254	340–362; 412–435; 504–528; 583–605	662–1096	1244–1541	—	—	—
			—	XP_012555367.1	1746	4–36; 41–70; 74–103; 162–192	340–362; 363–384; 387–409; 431–455	492–931	1072–1339	—	—	—
			—	XP_012560904.1	2121	353–382; 385–415	602–625; 626–649; 719–742; 743–769; 795–818; 868–892	937–1350	1479–1735	—	—	—
			—	XP_012557001.1	1643		335–358; 359–381; 387–410; 429–453	481–870	1004–1258	—	—	—
Amoebozoa	*D. discoideum*	Soil-dwelling amoeba	pats1	XP_645923.1	3184	—	1389–1413; 1414–1436; 1437–1460; 1465–1487; 1490–1512; 1539–1561; 1562–1584; 1585–1606; 1608–1630; 1631–1654; 1678–1701	1710–2127	2247–2511	—	2780–2820; 2900–2937; 2939–2977; 2980–3031	
Cryptophyta	*G. theta*	Cryptomonad algae	—	XP_005818488.1	302	—	—	—	44–299	—	—	—
			—	XP_005834159.1	682	—	—	—	179–432	—	—	**fh3**: 5–56; **FN3**: 67–155
Fungi	—	—	—	N	—	—	—	—	—	—	—	—
Plants	*R. argentea*	Silver myrtle	Lrrk1-like	XP_030537727.1	2699	353–382; 385–415	602–625; 626–649; 719–742; 743–769; 795–818; 868–892	937–1350	1479–1735	—	—	—

N means not found.

**FIGURE 5:**
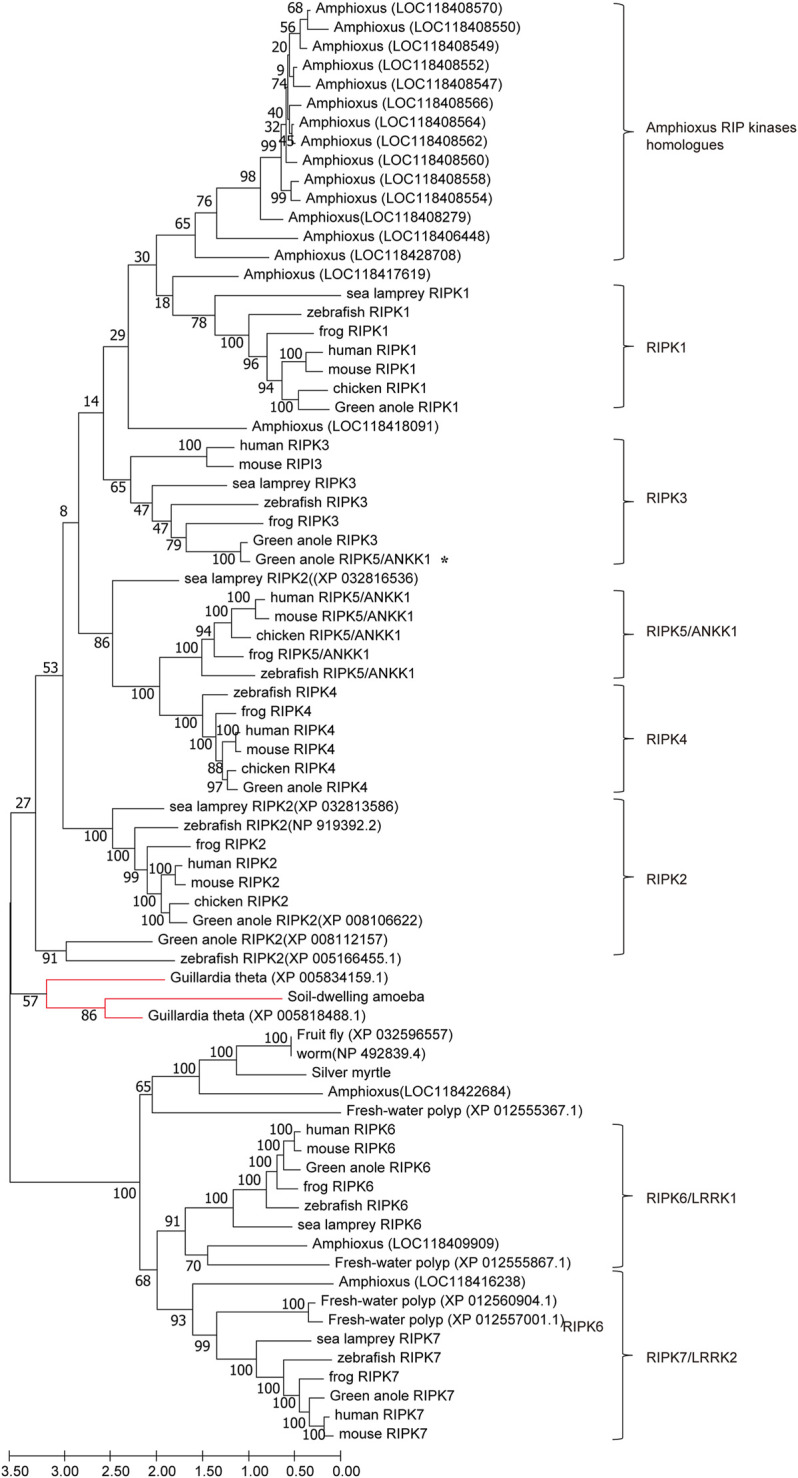
ML tree of RIPK1–7 across different species. The bootstrap percentage is shown on the interior branches. Entirely, the tree is divided into eight respective branches according to RIPK members and amphioxus, which is labeled next to the tree. Some vertebrate and invertebrate RIP kinase homologs are shown as species common names followed by GenBank accession numbers to distinguish genes with repeating or unclear names. The remaining homologs/orthologs are shown as species names followed by gene names. All sequence accession numbers used in this tree refer to Tables 2, 3. * indicates green anole ANKK1; the red line indicates sequences from *Guillardia theta* and soil-dwelling amoeba.

To examine the potential evolutionary relationships among *RIPK1–7* across different species, we used MEGA X to generate an ML tree using amino acid sequences from representative animals (Figure 5). Chicken Ripk6 and Ripk7 were not included in this tree because they decreased the overall bootstrap values. In total, the tree contains eight respective clades including each of the RIPK genes and amphioxus homologs. The content of each clade is consistent with established taxonomic relationships. The RIPK6 and RIPK7 clades are outgroups of the RIPK1–5 clades, which suggests that RIPK6 and RIPK7 may be more ancient than RIPK1–5. This is also supported by the lack of sequences from plants and invertebrates in the RIPK1–5 clades, except in amphioxus, which is representative of the transition between invertebrates and vertebrates. This phenomenon indicates RIPK1–5 may have arisen from one or more common ancestor and then have experienced two rounds of whole-genome duplication in amphioxus. The common ancestor of early-diverging vertebrates might be closer to the orthologues from cryptomonad algae (*G. theta*) and soil-dwelling amoeba, according to their location at the base of the RIPK1–5 clades. Meanwhile, amphioxus has 16 homologs in RIPK1–5 clades and three homologs in RIPK6 and RIPK7 clades, revealing amphioxus has undergone extensive lineage-specific duplication during two rounds of whole-genome duplication to produce the species with the most RIP kinase homologous present. In addition, the tree demonstrates that green anole ANKK1 (XP_008123542.1) in the NCBI database should be classified into RIPK3.

### Genomic organization

**FIGURE 6:**
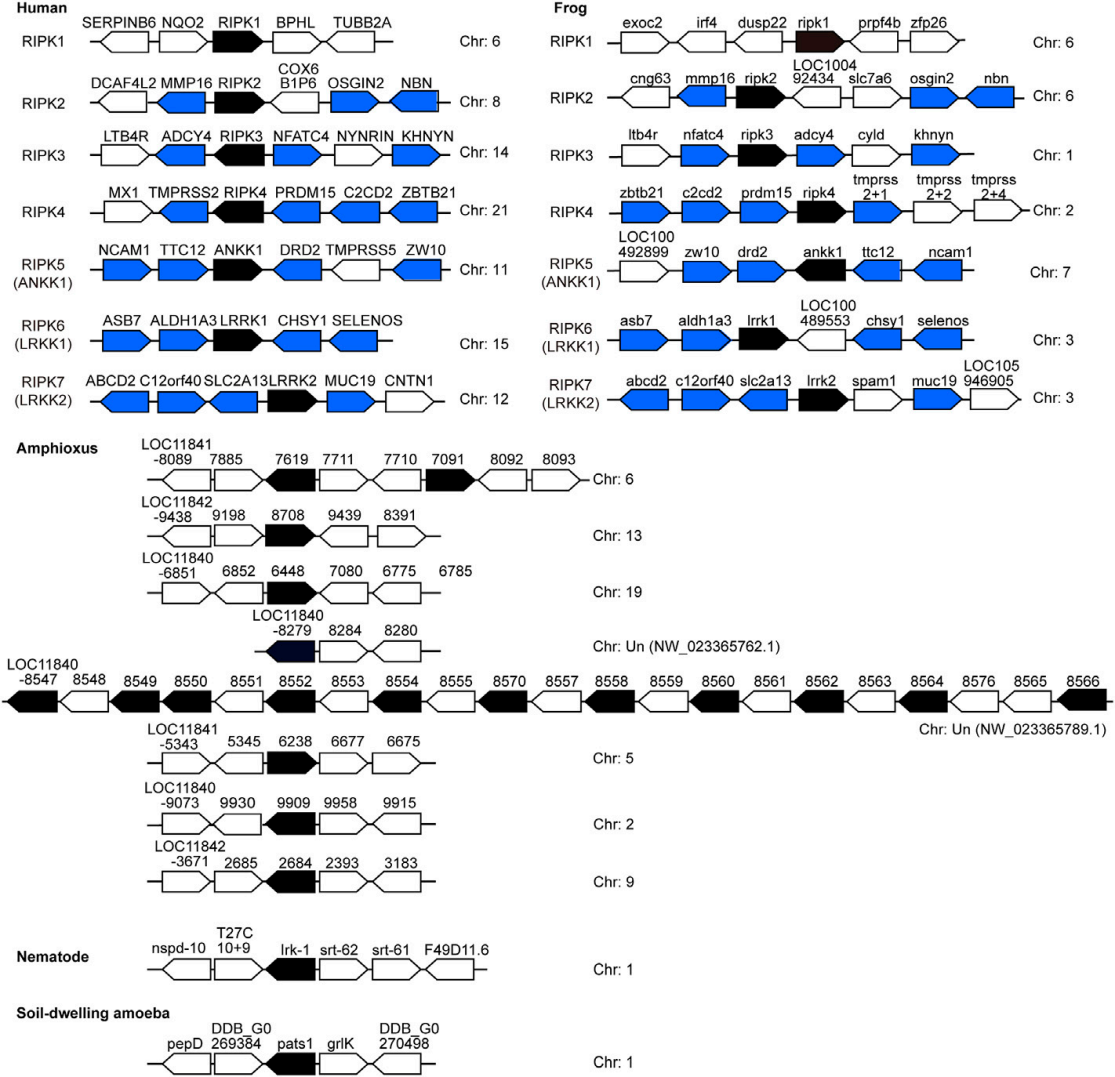
Chromosomal disposition of *RIP* kinase genes and their neighboring genes in the examined animals. Black blocks indicate putatively functional *RIP* kinases, whereas blue/white blocks represent other genes. Blue blocks indicate genes in common between human and frog. The distance between genes is not scaled to the locations of the chromosomal region. Arrows denote transcription orientation. Sequences refer to Tables 2, 3.

A comparison of the genomic organization of *RIPK1–7* homologs in vertebrates and invertebrates can provide clues to their evolutionary heritage. If different gene members originated historically from the duplication of a region in a common ancestor, other surrounding genes should have been duplicated at the same time (Hedges et al., 2004). Accordingly, we examined the genomic neighborhood surrounding *RIPK1–7* in mammals, amphibians, cephalochordates, nematomorpha, and amoebozoa (Figure 6).

Significantly, no similar surrounding genes were identified between vertebrates and invertebrates (amphioxus) as well as among *RIPK1–5* members from both human and frog. This phenomenon suggests the clear proto-orthologs of vertebrate *RIPK1–5* in amphioxus are difficult to be established. In human and frog, upstream and downstream genes of *RIPK2–7* positioned on different chromosomes demonstrate a strong conserved synteny, indicating a few intra-chromosomal and inter-chromosomal rearrangements for vertebrate *RIPK1–7* and RIP kinase evolution prior to the speciation events. However, amphioxus *RIP* kinase homologs are located in different chromosomes/regions: a single, two or eleven genes in a separate chromosome/region. This suggests an intra-chromosomal duplication, which is supported by the appearance of 14 copies clustered into one clade in Figure 5.

### Structural organization of RIPK1–7 from vertebrates and invertebrates

**FIGURE 7:**
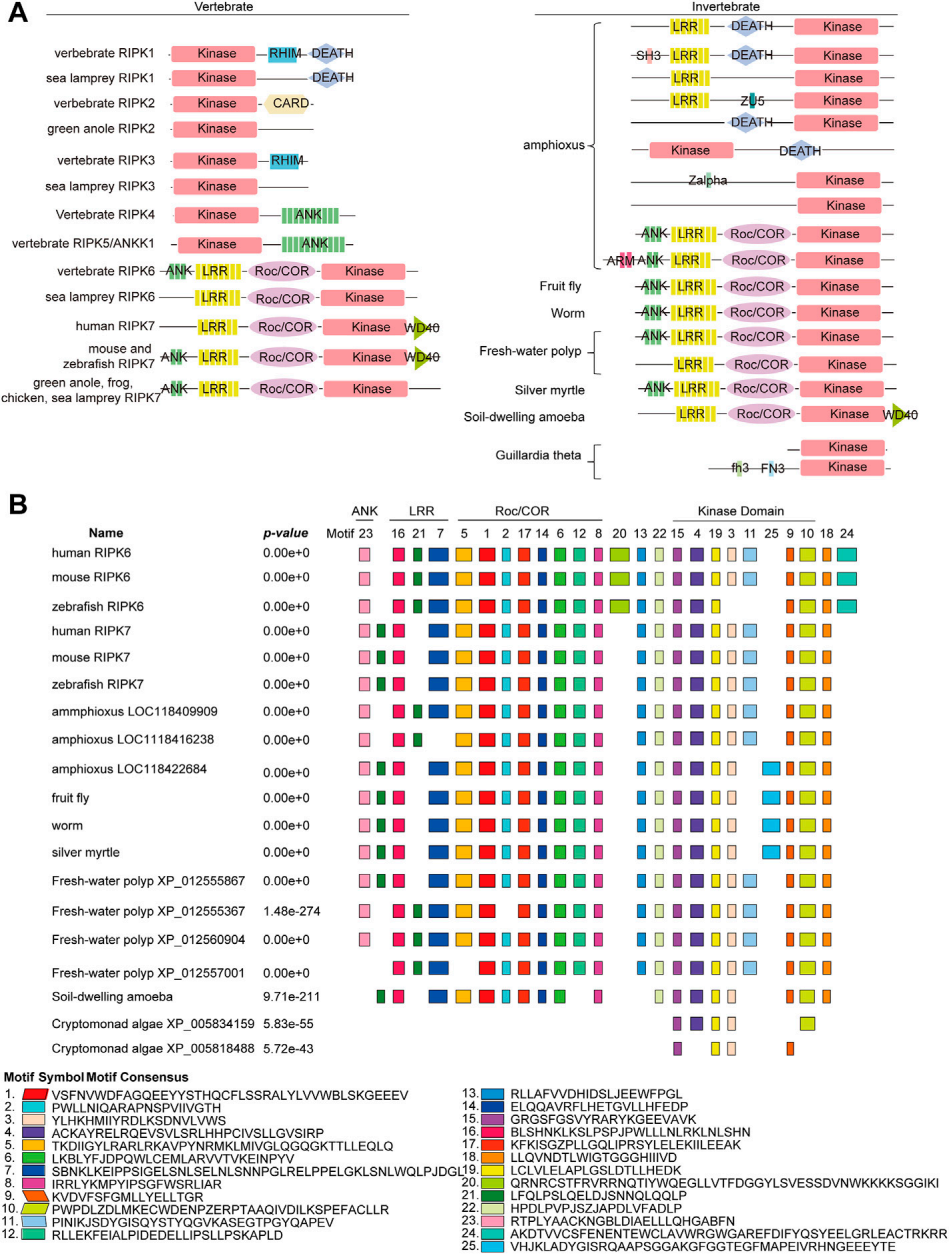
Conservation of the domain structure of RIPK1–7 from vertebrates and invertebrates **(A)** Diagram of the domain structure. **(B)** Motif composition of RIPK6 and RIPK7 with invertebrate RIP kinase by MEME analysis. Boxes of different colors indicate separate motifs. The best possible matched sequences for motifs are listed below the diagram.

Furthermore, the functional features of RIPK1–7 in vertebrates and invertebrates were identified by SMART as follows: (1) A Kinase domain is present in all RIPK homologs. (2) RHIM and CARD domain are only identified in vertebrate RIPK1, RIPK3, and RIPK2, which indicates that functions regulated by CARD and RHIM domain arose in vertebrates. (3) The combination of ANK and a Kinase domain is observed in vertebrate RIPK4 and RIPK5, while the combination of ANK-LRR-Roc/COR-Kinase is detected in both vertebrate RIPK6 and RIPK7 and invertebrates. (4) Many unique compositions of domain organization occur in amphioxus, such as LRR-Kinase, LRR-DEATH-Kinase, and DEATH-Kinase, as well as new small motif SH3, ZU5, and Zalpha, implying RIP kinase gene family in amphioxus has undergone gene conversion to produce the proto-ortholog of vertebrate RIPK1–5 (Figure 7A).

Taking into account the fact that the combination of ANK-LRR-Roc/COR-Kinase is the ancestral architecture of RIPK6 and RIPK7, but its functions are still unclear, we attempted to explore new smaller functional motifs in homologs in RIPK6 and RIPK7 clades using the MEME server. As shown in Figure 7B, eight conserved motifs (motif 3, 4, 9, 10, 11, 15, 19, 25) are detected in the Kinase domain, eight conserved motifs (motif 1, 2, 5, 6, 8, 12, 14, 17) in the Roc/COR domain, three motifs (motif 7, 16, 21) in the LRR domain, and one motif (Koeneke et al., 2020) in ANK. In particular, motif 15 and 19 are highly conserved in Kinase, as well as motif 1, 6, 8, 14 and 17 in Roc/COR domain, and motif 16 and 21 in LRR. Interestingly, motif 13, 22 and 18 are highly conserved, although they are located outside of functional domains. Motif 20 and 24 are only present in vertebrate RIPK6, which suggests they might be specifically involved in distinct vertebrate immune responses. Motif 25 is only observed in invertebrates, indicating it might be unique to invertebrate immune responses.

### RIPK3 loss in birds, snakes and early-diverging mammals

**FIGURE 8:**
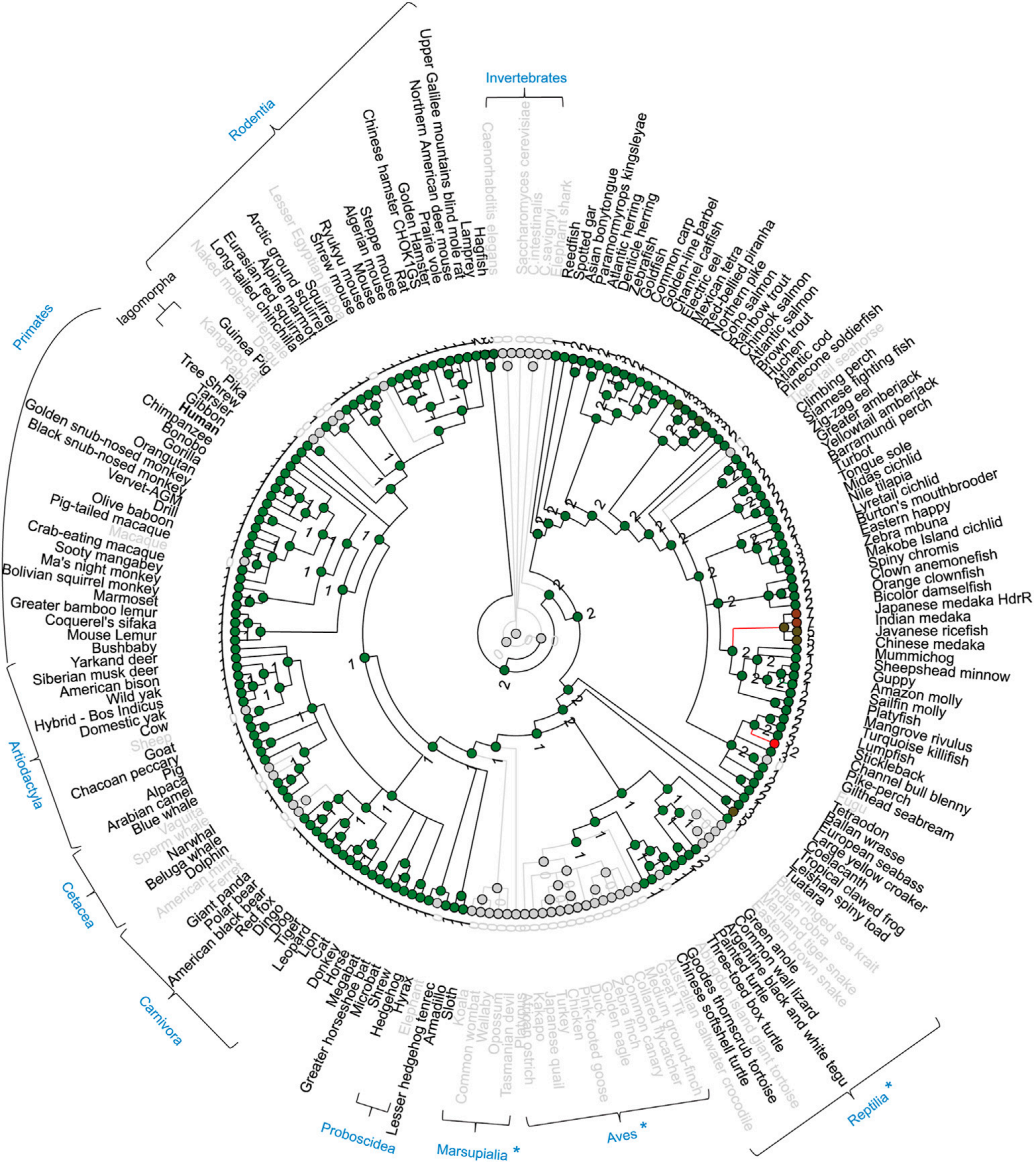
Gene gain/loss tree of *RIPK3* homologs of different species. The tree is retrieved from Comparative Genomics in the Ensembl database. The black line indicates no significant change. The red line indicates significant expansion. The green line indicates significant contraction. Species in gray are species with no gene. The number of homologs is labeled in the nodes. Gray circles indicate nodes with 0 number. { indicates species taxonomy.

In the RIPK3 clade, RIPK3 loss was observed in chicken. Interestingly, we also found *RIPK3* loss in many other species, including the complete class of Aves (all 13 birds), the infraclass of Reptilia (4 kinds of snake, tortoise, crocodile), some early-diverging mammals (platypus, koala, common wombat, wallaby, opossum, Tasmanian devil) (Figure 8). A common characteristic of these animals with RIPK3 loss is that they undergo torpor, a physiological phenomenon during cold environmental conditions, when they slow their body metabolism and lower body temperature overnight or the whole season (Dondelinger et al., 2016). Torpor can be considered as a cold ischemia condition followed by reperfusion when body metabolism and temperature are restored (Bogren et al., 2014). Meanwhile, accumulating evidences indicate gene loss in birds is generally associated with physiological features, such as hyperglycemia, high metabolic rate, non-shivering thermogenesis loss, and low glomerular filtration rate (Xiong and Lei, 2021). RIPK3 may be involved in ischemia-reperfusion injury or high metabolic rate through glucose homeostasis, and therefore RIPK3 loss can represent an evolutionary adaptation to the physiological characteristics of torpor (Dondelinger et al., 2016).

Moreover, possibly because of the incompleteness of the genome sequencing or lineage-specific gene loss/evolution in mammals, RIPK3 loss was also observed in macaque *Macaca mulatta* (Primates), kangaroo rat *Dipodomys ordii* (Rodentia), rabbit *Oryctolagus cuniculus* (Lagomorpha), ferret *Mustela putorius furo* (Carnivora), sperm whale *Physeter catodon* (Cetacea), sheep *Ovis aries* (Artiodactyla), and elephant *Loxodonta africana* (Proboscidea).

## Discussion

### Perplexing information on RIPK4–7 in reported studies

Currently, there is some inconsistent information regarding which gene can be considered as *RIPK5*. Zha et al., 2004 found that DSTYK overexpression can induce caspase-dependent and -independent cell death and DNA fragmentation in 293 cell line and thereby proposed DSTYK as RIPK5 (Zha et al., 2004). Cuny and Degterev 2021 identified that DSTYK/SgK496 had homology to RIPK4, and ANKK1 was most similar to *RIPK1* based on the phylogenic tree of the Kinase domain. Thus, both *DSTYK* and *ANKK1* could be considered as RIPK members (Cuny and Degterev, 2021). Yet the majority of public databases describe DSTYK as RIPK5. However, since the analysis of the human kinome by Manning *et al.* in 2002, RIP kinase researchers have referred to ANKK1/SgK288 as RIPK5 (Manning et al., 2002). In this study, our phylogenetic trees of full-length amino acid sequences (Figure 3B) and Kinase domain (Figure 4A) provide evidence that DSTYK is clearly distinct from RIPK1–7, in contrast to ANKK1.

Meanwhile, it is worth noting that the domain organization of human *RIPK6* and *RIPK7* differs in different papers. That is, RIPK6 is shown with a WD40 motif by Humphries et al., 2015 and He and Wang, 2018, but without WD40 in Zhang et al., 2009 and Meylan and Tschopp, 2005. RIPK7 is shown with an N-terminal ANK motif by He and Wang, 2018, whereas an N-terminal ANK and ARM (Armadillo) motif is attributed by Humphries et al., 2015, and no ANK is shown by Zhang et al., 2009 and Meylan and Tschopp, 2005. Our Figure 1A with domains predicted by SMART, supports Zhang et al., 2009 and Meylan and Tschopp, 2005 (Meylan and Tschopp, 2005; Zhang et al., 2010; Humphries et al., 2015; He and Wang, 2018).

Overall, future studies would benefit from a clear description of *RIPK4–7* to aid in the dissemination of results between different research groups.

### Potential function of RIPK4 and RIPK5 (ANKK1) in immune responses

Given the negligible expression of *Ripk4* in immune cells (Figure 2A), it is not surprising that the direct role of *Ripk4* in immune responses has not been studied. Nevertheless, the close evolutionary relationships of the expression patterns of RIPK4 and RIPK1 in human organs (Figure 1C), and the fact that 10 of 12 critical residues are homologous between RIPK1 and RIPK4 (Figure 4B), demonstrates that RIPK4 may be involved in NF-κB and MAPK activation like RIPK1 in non-immune cell types. This is supported by the observation that *RIPK4* overexpression can activate NF-κB and MAPK in 293 T cells and can induce pro-inflammatory cytokine interleukin-8 and chemokine CCL5 and CXCL11 in human oral keratinocytes (Meylan et al., 2002; Kwa et al., 2016).

Nevertheless, the domain structure of ANKK1 is highly similar to RIPK4 with an overall identity of 35% (Meylan and Tschopp, 2005), which suggests that ANKK1 might have a function similar to RIPK4 in the induction of pro-inflammatory cytokines (Meylan et al., 2002; Shin et al., 2008). ANKK1 is dominantly expressed in brain (Supplementary Figure S1), which is consistent with the polymorphism Taq1A that leads to ANKK1 reduced stability being associated with schizophrenia (Habibzadeh et al., 2021). However, immune cell expression by ImmGen (Figure 2A) does not include immune cells from the brain, such as microglia. Therefore, the potential role of ANKK1 in inflammation could not be excluded, especially given the significant association between inflammation and neuropsychiatric disorders (Williams et al., 2022).

### Potential function of RIPK6 and RIPK7 in immune responses

*RIPK6* and *RIPK7* homologs have been identified in early-diverging invertebrate worm, fruitfly and fresh-water polyp, and even plants (Table 3; Figure 5 and Figure 7A). This distinguishes them from *RIPK1–3*, revealing RIPK6 and RIPK7 might be involved in some evolutionarily conserved signaling pathways, such as cadmium signal transduction pathways in cell homeostasis and the MAPK pathway in innate immune responses (Ausubel, 2005; Chmielowska-Bąk and Deckert, 2012). At the same time, the dominant expression of *Ripk7* in innate immune cells and its absence in T cells reveals that *Ripk7* may participate preferentially in innate immune responses rather than adaptive immune responses (Figure 2A). RIPK7 mutations and kinase activity play very important roles in the pathogenesis of PD (Berwick et al., 2019; Seegobin et al., 2020), whereas *RIPK7* is expressed at low levels in neurons but at high levels in immune cells. Significantly, a recent genome-wide association study demonstrated that genetic polymorphisms in *RIPK7* are associated with autoimmune disease multibacillary leprosy and inflammatory bowel disease (Zhang et al., 2009; Hui et al., 2018). Meanwhile, *RIPK7* contributes to cytokine production in response to a limited group of bacterial pathogens, but the outcomes and mechanisms mediated by *RIPK7* for viral or fungal pathogens are poorly characterized (Eng et al., 2021). Future research on *RIPK6* and *RIPK7* may shift from neuronal toxicity to inflammation and cell death.

## Data Availability

Publicly available datasets were analyzed in this study. Further inquiries can be requested from the corresponding author.
