# Supplementary figures and images for: Comparative and evolutionary analysis of RIP kinases in immune responses
Source: Front Genet. 2022 Oct 3;13:796291. doi: 10.3389/fgene.2022.796291 (PMC9573974; doi:10.3389/fgene.2022.796291)

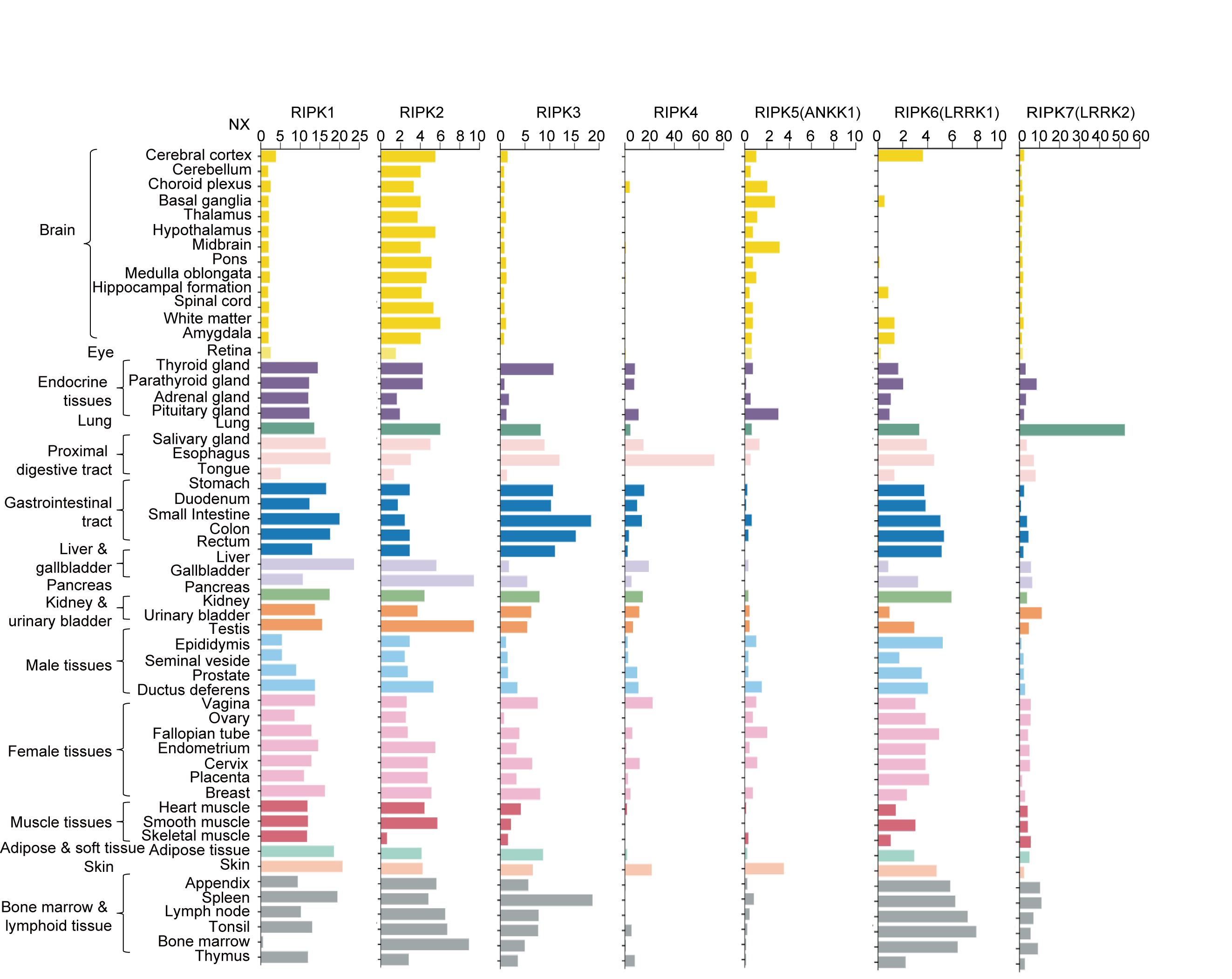

Supplement: Supplementary file 1 [file Image1.TIF]
