# Supplementary material for: Comparative and evolutionary analysis of RIP kinases in immune responses
Source: Front Genet. 2022 Oct 3;13:796291. doi: 10.3389/fgene.2022.796291 (PMC9573974; doi:10.3389/fgene.2022.796291)
Supplement: Supplementary file 2 [file DataSheet1.docx]

**Supplementary Material**

**Supplementary Figure 1.** Expression profiles of *RIPK1****–****7* in different tissues by the consensus data sets of HPA, GTEx, and FANTOM5 in the Human Protein Atlas. NX means normalized expression.

| **Table 1. The overall sequence information of paralogues of human *RIPK1–7* in Ensembl genome database** | | | | | | | | | | |
| --- | --- | --- | --- | --- | --- | --- | --- | --- | --- | --- |
| **Gene** | **Accession Numbers** | **Length (aa)** | **Position of Kinase domain** | **Position of DEATH domain** | **Position of ANK domain** | **Position of LRR domain** | **Position of Roc/COR/RBD domain** | **Position of C1 domain** | **Position of PDZ domain** | **Position of other domain** |
| ARAF | NP_001645.1 | 606 | 310-568 |  |  |  | 19-91 | 99-144 |  |  |
| BRAF | NP_004324.2 | 766 | 457-716 |  |  |  | 155-227 | 235-280 |  |  |
| FPTG-TNNI3K | NP_001106279.3 | 936 | 564-820 |  | 167-197; 201-230; 234-263; 267-296; 300-331; 335-366; 370-401; 405-436; 440-469; 482-511 |  |  |  |  |  |
| ILK | NP_001014794.1 | 452 | 195-445 |  | 33-62; 66-95; 99-128 |  |  |  |  |  |
| KSR1 | NP_055053.1 | 759 | 476-741 |  |  |  |  | 211-254 |  |  |
| KSR2 | NP_775869.4 | 950 | 666-928 |  |  |  |  | 411-456 |  | **KSR1-SAM:**24-152 |
| LIMK1 | NP_002305.1 | 647 | 339-604 |  |  |  |  | 339-604 | 176-258 | **LIM**:24-75; 83-137 |
| LIMK2 | NP_001026971.1 | 686 | 310-572 |  |  |  |  |  | 140-218 | **LIM**:7-42; 50-103; **PP1-inhibitor**: 577-686 |
| MAP3K9 | NP_149132.2 | 1118 | 144-403 |  |  |  |  |  |  | **SH3**: 55-115 |
| MAP3K10 | XP_011525283.1 | 962 | 98-365 |  |  |  |  |  |  | **SH3**: 19-80 |
| MAP3K11 | XP_011525283.1 | 847 | 117-376 |  |  |  |  |  |  | **SH3**: 44-104 |
| MAP3K13 | NP_001229243.1 | 966 | 168-407 |  |  |  |  |  |  |  |
| MAP3K20 | NP_057737.2 | 800 | 16-259 |  |  |  |  |  |  | **KSR1-SAM:**336-410 |
| MAP3K21 | NP_115811.2 | 1036 | 124-398 |  |  |  |  |  |  | **SH3**: 41-101 |
| MLKL | NP_689862.1 | 471 | 213-466 |  |  |  |  |  |  |  |
| MOS | NP_005363.1 | 344 | 61-337 |  |  |  |  |  |  |  |
| RAF1 | NP_001341618.1 | 668 | 369-628 |  |  |  | 56-131 | 139-184 |  |  |
| TNNI3K | NP_057062.1 | 835 | 463-719 |  | 66-96; 100-129; 133-162; 166-195; 199-230; 234-165; 269-300; 304-335; 339-368; 381-410 |  |  |  |  |  |
| TESK1 | NP_006276.2 | 626 | 57-314 |  |  |  |  |  |  |  |
| TESK2 | NP_009101.2 | 571 | 60-309 |  |  |  |  |  |  |  |
| ANKK1 | NP_848605.1 | 765 | 25-285 |  | 361-390; 394-423; 427-456; 460-489; 493-522; 526-555; 559-588; 592-621; 625-654; 658-687; 691-720 |  |  |  |  |  |
| NRBP1 | NP_848605.1 | 535 | 81-324 |  |  |  |  |  |  |  |
| NRBP2 | NP_848659.2 | 501 | 55-306 |  |  |  |  |  |  |  |
| WNK1 | NP_061852.3 | 2382 | 221-479 |  |  |  |  |  |  |  |
| WNK2 | NP_001269323.1 | 2297 | 195-451 |  |  |  | 474-537 |  |  |  |
| WNK3 | NP_065973.2 | 1800 | 147-405 |  |  |  | 426-489 |  |  |  |
| WNK4 | NP_115763.2 | 1243 | 174-430 |  |  |  |  |  |  | **OSR1-C:**453-515 |
| ERBIN | NP_001240626.1 | 1412 |  |  |  | 48-68; 91-114; 137-159; 160-182; 183-205; 206-228; 229-252; 253-274; 275-298; 321-344; 345-366; 367-389 |  |  |  |  |
| LRRC1 | NP_060684.4 | 524 |  |  |  | 35-57; 58-80; 83-104; 104-123; 127-146; 150-172; 173-195; 196-218; 219-241; 242-264; 265-288; 288-307; 311-334; 336-357; 358-380 |  |  |  |  |
| LRRC2 | NP_078788.2 | 371 |  |  |  | 143-165; 166-189; 236-258; 259-282 |  |  |  |  |
| LRRC7 | NP_001317564.1 | 1495 |  |  |  | 53-73; 96-108; 142-164; 165-187; 188-210; 211-233; 234-257; 258-279; 280-303; 326-349; 372-394 |  |  |  |  |
| LRRC8A | NP_001120716.1 | 810 |  |  |  | 590-613; 614-636; 638-660; 661-684; 685-706; 707-730; 731-751; 753-776 |  |  |  | Pannexin_like: 1-340 |
| LRRC8B | NP_001127948.1 | 803 |  |  |  | 509-536; 584-607; 609-630; 632-654; 655-678; 679-700; 701-724; 747-770 |  |  |  | Pannexin_like: 1-334 |
| LRRC8C | NP_115646.3 | 803 |  |  |  | 588-611; 613-635; 636-658; 659-682; 684-703; 705-728; 751-774 |  |  |  | Pannexin_like: 1-338 |
| LRRC8D | NP_001127951.1 | 858 |  |  |  | 657-680; 682-704; 705-728; 729-750; 751-774; 775-796; 797-820 |  |  |  | Pannexin_like: 1-384 |
| LRRC8E | NP_001255213.1 | 796 |  |  |  | 604-628; 629-651; 652-675; 676-697; 698-721; 722-743; 744-767 |  |  |  | Pannexin_like: 1-331 |
| LRRC10 | NP_963844.2 | 277 |  |  |  | 51-73; 74-97; 120-143; 166-189 |  |  |  |  |
| LRRC10B | NP_001138549.1 | 292 |  |  |  | 43-65; 66-87; 89-111; 135-156; 158-181 |  |  |  |  |
| LRRC18 | NP_001006940.3 | 261 |  |  |  | 49-71; 72-95; 120-142; 143-166 |  |  |  |  |
| LRCC27 | NP_001137229.1 | 530 |  |  |  | 66-89; 90-113; 114-136 |  |  |  |  |
| LRCC28 | NP_001308604.1 | 367 |  |  |  | 40-63; 64-86; 87-109; 110-132; 133-156; 179-202 |  |  |  |  |
| LRCC30 | NP_001099051.1 | 301 |  |  |  | 70-92; 93-115; 116-139; 140-161; 162-185; 208-230; 231-254 |  |  |  |  |
| LRCC39 | NP_001243314.1 | 339 |  |  |  | 105-127; 128-151; 175-197; 198-220; 221-243; 244-267 |  |  |  |  |
| LRCC40 | NP_060238.3 | 602 |  |  |  | 81-100; 104-126; 127-149; 150-172; 173-195; 196-218; 219-241; 242-264; 288-310; 311-334; 335-356; 471-493; 494-517; 541-564 |  |  |  |  |
| LRCC58 | NP_001093148.1 | 371 |  |  |  | 44-66; 67-90; 119-141; 142-164; 165-187; 188-210; 211-234 |  |  |  |  |
| LRCC69 | NP_001123362.1 | 347 |  |  |  | 36-58; 59-81; 82-105; 106-128; 129-150; 152-174; 175-198; 200-221 |  |  |  |  |
| LRCH1 | NP_001157683.2 | 763 |  |  |  | 119-141; 142-165; 187-209; 210-233; 255-278 |  |  |  | **CH：**613-722 |
| LRCH2 | NP_065922.3 | 765 |  |  |  | 133-155; 156-179; 181-201; 224-247; 269-292 |  |  |  |  |
| LRCH3 | NP_001350816.1 | 803 |  |  |  | 104-126; 127-150; 172-194; 195-218; 240-263 |  |  |  | **CH：**658-762 |
| LRCH4 | NP_002310.2 | 683 |  |  |  | 90-112; 158-180; 226-249 |  |  |  | **CH：**540-645 |
| LRRD1 | NP_001155000.1 | 860 |  |  |  | 187-209; 256-278; 279-301; 302-324; 371-394; 395-416; 417-440; 486-509; 532-555; 650-671; 673-695; 696-719; 721-742 |  |  |  |  |
| LRRIQ4 | NP_001073929.1 | 560 |  |  |  | 47-69; 70-92; 93-116; 117-140; 141-164; 187-209; 210-233; 234-255; 256-279; 302-324; 325-347; 348-371; 397-419; 420-443; 444-466; |  |  |  | **IQ :503-525** |
| MFHAS1 | NP_004216.2 | 1052 |  |  |  | 62-85; 86-109; 110-129; 134-156; 180-202; 203-225; 226-248; 272-294; 295-317; 318-338; 341-364 | 411-541 |  |  |  |
| PHLPP1 | NP_919431.2 | 1717 |  |  |  | 692-713; 713-732; 736-758; 759-781; 782-805; 830-853; 893-912; 916-939; 939-958; 963-984; 985-1004; 1035-1054; 1059-1082 |  |  |  | **PH :**537-638;**PP2Cc** :1165-1420 |
| PHLPP2 | NP_055835.2 | 1323 |  |  |  | 298-317; 321-343; 344-366; 367-386; 459-482; 501-520; 524-543; 547-566; 572-592; 593-612; 619-644; 643-662; 667-690; 690-709; 712-736 |  |  |  | **PP2Cc :**775-1031 |
| PIDD1 | NP_665893.2 | 910 |  | 778-873 |  | 124-146; 147-169; 170-192; 193-215; 216-238; 239-261; 262-285 |  |  |  | **ZU5:**323-417;457-545;**Peptidase-S68** :421-453 |
| SCRIB | NP_874365.3 | 1655 |  |  |  | 58-80; 81-104; 127-149; 150-172; 173-195; 219-241; 242-265; 335-356; 357-380 |  |  | 736-815; 870-950; 1012-1093; 1109-1192 |  |
| SHOC2 | NP_001311265.1 | 582 |  |  |  | 122-144; 145-167; 168-190; 191-213; 214-235; 237-260; 283-306; 307-329; 330-353; 354-377; 401-423; 424-446; 447-469; 470-492; 493-514; 516-540 |  |  |  |  |

**Table 2. The *RIPK1–7* homologs from representative animals in vertebrates**

| **Gene** | **Species** | **Common Name** | **Accession numbers** | **Identities to human RIPK1** | **Length (aa)** | **Position of Kinase** | **Position of RHIM** | **Position of DEATH** |  |  |
| --- | --- | --- | --- | --- | --- | --- | --- | --- | --- | --- |
| RIPK1 | *H. sapiens* | Human | NP_001341859.1 |  | 671 | 17-285 | 504-549 | 573-669 |  |  |
|  | *M. musculus* | Mouse | NP_001346926.1 | 69.80% | 656 | 18-286 | 480-538 | 558-654 |  |  |
|  | *G. gallus* | Chicken | NP_989733.2 | 48.60% | 658 | 13-281 | 487-536 | 560-656 |  |  |
|  | *A. carolinensis* | Green anole | XP_003224434.1 | 48.50% | 699 | 13-279 | 520-574 | 595-691 |  |  |
|  | *X. tropicalis* | Frog | NP_001072503.1 | 42.90% | 669 | 17-288 | 474-542 | 564-661 |  |  |
|  | *D. rerio* | zebrafish | NP_001036815.1 | 39.00% | 661 | 15-284 | 480-534 | 558-654 |  |  |
|  | *P. marinus* | Sea lamprey | XP_032813622.1 | 31.90% | 744 | 14-293 |  | 651-743 |  |  |
| **Gene** | **Species** | **Common Name** | **Accession numbers** | **Identities to human RIPK2** | **Length (aa)** | **Position of Kinase** | **Position of CARD** | **Position of DEATH** |  |  |
| RIPK2 | *H. sapiens* | Human | NP_003812.1 |  | 540 | 18-289 | 435-526 |  |  |  |
|  | *M. musculus* | Mouse | NP_620402.1 | 84.30% | 539 | 18-290 | 434-522 |  |  |  |
|  | *G. gallus* | Chicken | NP_001026114.1 | 63.70% | 574 | 27-295 | 479-563 |  |  |  |
|  | *A. carolinensis* | Green anole | XP_008106622.1 | 64.40% | 560 | 38-307 | 464-548 |  |  |  |
|  |  |  | XP_008112157.1 | 35.00% | 392 | 1-215 |  |  |  |  |
|  | *X. tropicalis* | Frog | XP_002939201.2 | 52.80% | 553 | 47-320 | 462-553 |  |  |  |
|  | *D. rerio* | zebrafish | NP_919392.2 | 51.30% | 584 | 28-300 | 470-556 |  |  |  |
|  |  |  | XP_005166455.1 | 27.20% | 513 | 21-264 | 424-503 |  |  |  |
|  | *P. marinus* | Sea lamprey | XP_032813586.1 | 51.00% | 664 | 18-287 | 573-660 |  |  |  |
|  |  |  | XP_032816536.1 | 34.50% | 545 | 26-378 |  | 452-541 |  |  |
| **Gene** | **Species** | **Common Name** | **Accession numbers** | **Identities to human RIPK3** | **Length (aa)** | **Position of Kinase** | **Position of RHIM** |  |  |  |
| RIPK3 | *H. sapiens* | Human | NP_006862.2 |  | 518 | 21-283 | 417-468 |  |  |  |
|  | *M. musculus* | Mouse | NP_064339.2 | 60.40% | 486 | 22-288 | 408-458 |  |  |  |
|  | *G. gallus* | Chicken | N |  |  |  |  |  |  |  |
|  | *A. carolinensis* | Green anole | XP_003223896.2 | 36.70% | 488 | 16-290 | 434-478 |  |  |  |
|  | *X. tropicalis* | Frog | XP_002934332.3 | 36.30% | 514 | 13-276 | 401-439 |  |  |  |
|  | *D. rerio* | zebrafish | XP_001343827.1 | 38.50% | 433 | 19-287 | 341-403 |  |  |  |
|  | *P. marinus* | Sea lamprey | XP_032816533.1 | 38.00% | 634 | 81-347 |  |  |  |  |
| **Gene** | **Species** | **Common Name** | **Accession numbers** | **Identities to human RIPK4** | **Length (aa)** | **Position of Kinase** | **Position of ANK** |  |  |  |
| RIPK4 | *H. sapiens* | Human | NP_065690.2 |  | 784 | 22-283 | 437-466; 470-499; 503-532; 536-565; 569-599; 603-632; 636-665; 669-698; 702-732; 734-763 |  |  |  |
|  | *M. musculus* | Mouse | NP_076152.2 | 90.50% | 786 | 23-283 | 439-468; 472-501; 505-534; 538-567; 571-601; 605-634; 638-667; 671-700; 704-734; 736-765 |  |  |  |
|  | *G. gallus* | Chicken | XP_004934622.2 | 77.20% | 789 | 23-283 | 436-485; 489-498; 502-531; 535-564; 568-598; 602-631; 635-664; 668-697; 701-729; 733-762 |  |  |  |
|  | *A. carolinensis* | Green anole | XP_003219008.1 | 76.10% | 788 | 22-283 | 434-463; 467-496; 500-529; 533-562; 566-596; 600-629; 633-662; 666-695`; 699-727; 731-760 |  |  |  |
|  | *X. tropicalis* | Frog | XP_002941332.1 | 71.40% | 717 | 24-283 | 438-467; 471-500; 504-533; 537-566; 570-600; 604-633; 637-666; 670-699 |  |  |  |
|  | *D. rerio* | zebrafish | NP_998243.1 | 62.10% | 820 | 23-283 | 433-462; 466-496; 500-529;533-562; 566-596; 600-629; 633-662; 666-695; 699-728;733-762 |  |  |  |
|  | *P. marinus* | Sea lamprey | N |  |  |  |  |  |  |  |
| **Gene** | **Species** | **Common Name** | **Accession numbers** | **Identities to human RIPK5** | **Length (aa)** | **Position of Kinase** | **Position of ANK** |  |  |  |
| RIPK5 (ANKK1) | *H. sapiens* | Human | NP_848605.1 |  | 765 | 25-285 | 361-390; 394-423; 427-456; 460-489; 493-522; 526-555; 559-588; 592-621; 625-654; 658-687; 691-720 |  |  |  |
|  | *M. musculus* | Mouse | NP_001363880.1 | 79.00% | 746 | 35-297 | 370-399; 403-432; 436-465; 469-498; 502-531; 535-564; 568-597; 601-630; 634-663; 667-696; 700-729 |  |  |  |
|  | *G. gallus* | Chicken | XP_003642663.2 | 61.50% | 830 | 70-332 | 414-443; 447-476; 480-509; 513-542; 546-575; 579-608; 612-641; 645-674; 678-707; 711-740; 744-773; 777-806 |  |  |  |
|  | *A. carolinensis* | Green anole | XP_008123542.1 | 38.90% | 379 (partial) | 16-290 |  |  |  |  |
|  | *X. tropicalis* | Frog | XP_002937872.1 | 53.20% | 766 | 30-289 | 358-387; 391-420; 424-453; 457-486; 490-519; 523-552; 556-585; 589-618; 622-651; 655-684; 688-717; 721-749; 765-794; 798-827 |  |  |  |
|  | *D. rerio* | zebrafish | NP_001124137.1 | 41.90% | 733 | 30-282 | 347-376; 380-409; 413-442; 446-475; 480-509; 513-542; 546-575; 579-608; 612-641; 645-674 |  |  |  |
|  | *P. marinus* | Sea lamprey | N |  |  |  |  |  |  |  |
| **Gene** | **Species** | **Common Name** | **Accession numbers** | **Identities to human RIPK6** | **Length (aa)** | **Position of ANK** | **Position of LRR** | **Position of Roc/COR** | **Position of Kinase** |  |
| RIPK6 (LRRK1) | *H. sapiens* | Human | NP_078928.3 |  | 2015 | 119-148; 152-182; 193-223 | 278-300; 301-324; 328-351; 379-401; 403-427; 472-493; 548-569; 570-594 | 623-1046 | 1243-1520 |  |
|  | *M. musculus* | Mouse | NP_666303.3 | 88.80% | 2014 | [86-116; 119-148; 162-182; 193-223](http://www.uniprot.org/uniprot/Q3UHC2) | 278-300; 301-325; 328-351; 379-401; 403-427; 472-493; 548-569; 570-594 | 625-1046 | 1244-1520 |  |
|  | *G. gallus* | Chicken | NP_001376322 | 77.6% | 1998 | 76-105; 109-138; 142-172; 182-212 | 267-286; 291-310; 318-337; 369-388; 392-412; 441-462; 462-481; 537-556; 560-581; | 630-1036 | 1231-1509 |  |
|  | *A. carolinensis* | Green anole | XP_008116735.1 | 73.50% | 2000 | 76-106; 109-139; 142-172; 183-212 | 268-290;291-313; 318-341; 369-392; 393-417; 462-483; 536-558; 560-584 | 630-1034 | 1233-1510 |  |
|  | *X. tropicalis* | Frog | XP_012815031.2 | 67.50% | 2003 | 110-139; 143-173; 184-213 | 270-289; 294-313; 321-340; 371-390; 395-414; 464-488; 539-558; 562-582 | 632-1038 | 1233-1512 |  |
|  | *D. rerio* | zebrafish | XP_021333791.1 | 56.90% | 2007 | 107-137; 140-169; 173-201; 214-243 | 298-317; 322-346; 349-368; 400-423; 424-443; 448-464; 472-489; 493-512; 566-585; 613-636 | 659-1067 | 1267-1498 |  |
|  | *P. marinus* | Sea lamprey | XP_032806825.1 | 41.80% | 1825 |  | 59-78; 83-102; 132-151; 161-184; 185-204; 231-250; 254-273; 339-358; 362-382 | 434-883 | 1080-1361 |  |
| **Gene** | **Species** | **Common Name** | **Accession numbers** | **Identities to human RIPK7** | **Length (aa)** | **Position of ANK** | **Position of LRR** | **Position of Roc/COR** | **Position of Kinase** | **Position of WD40** |
| RIPK7 (LRRK2) | *H. sapiens* | Human | NP_940980.4 |  | 2527 |  | 1010-1033; 1034-1057; 1082-1105; 1128-1151; 1195-1219; 1244-1266; 1267-1291 | 1319-1740 | 1882-2132 | 2231-2276 |
|  | *M. musculus* | Mouse | NP_080006.3 | 86.60% | 2527 | 708-737; 770-800 | 1010-1033; 1034-1057; 1082-1105; 1128-1151; 1195-1219; 1244-1266; 1267-1291 | 1336-1740 | 1882-2132 | 2231-2274; 2401-2438 |
|  | *G. gallus* | Chicken | NP_001274122.2 | 72.60% | 2557 | 736-765; 798-828 | 1039-1062; 1063-1082;1111-1130; 1135-1154; 1157-1176; 1181-1200; 1201-1220; 1224-1243; 1274-1291; 1296-1316 | 1364-1769 | 1904-2161 |  |
|  | *A. carolinensis* | Green anole | XP_008109800.1 | 72.10% | 2540 | 719-748; 781-811 | 1022-1045; 1046-1069; 1094-1116; 1140-1163; 1183-1206; 1207-1231; 1256-1279 | 1332-1752 | 1892-2144 |  |
|  | *X. tropicalis* | Frog | XP_002932250.3 | 62.40% | 2514 | 406-435; 705-734; 737-768 | 1002-1024; 1026-1045; 1074-1093; 1120-1143; 1187-1206; 1237-1255; 1259-1279 | 1329-1732 | 1867-2124 |  |
|  | *D. rerio* | zebrafish | NP_001188385.2 | 47.70% | 2556 | 735-764; 767-798 | 1026-1045; 1050-1073; 1098-1117; 1122-1141; 1144-1163; 1188-1207; 1211-1235; 1261-1279; 1283-1303 | 1351-1750 | 1889-2146 | 2215-2248; 2251-2296; 2359-2399 |
|  | *P. marinus* | Sea lamprey | XP_032810325.1 | 38.30% | 2533 | 736-765; 768-800 | 984-1004; 1008-1027; 1056-1078; 1080-1100; 1102-1122; 1146-1165; 1169-1193; 1219-1237; 1241-1264 | 1307-1710 | 1864-2120 |  |

“N” means not found.

**Table 3. The orthologue of RIP kinases from representative animals in invertebrates**

| **Phylum** | **Species** | **Common Name** | **Gene** | **Accession numbers** | **Length (aa)** | **Position of ANK** | **Position of LRR** | **Position of Roc/COR** | **Position of Kinase** | **Position of DEATH** | **Position of WD40** | **Position of Other domain** |
| --- | --- | --- | --- | --- | --- | --- | --- | --- | --- | --- | --- | --- |
| Choanozoa | *B. floridae* | Amphioxus | LOC118408564 | XP_035665266.1 | 254 |  |  |  | 1-225 |  |  |  |
|  |  |  | LOC118428708 | XP_035694742.1 | 458 |  |  |  | 156-415 |  |  | **Zalpha**: 1-63 |
|  |  |  | LOC118408549 | XP_035665251.1 | 344 |  |  |  | 58-319 |  |  |  |
|  |  |  | LOC118417619 | XP_035679122.1 | 539 |  |  |  | 17-280 | 448-539 |  |  |
|  |  |  | LOC118418091 | XP_035679812.1 | 705 |  |  |  | 20-284 | 614-705 |  |  |
|  |  |  | LOC118408554 | XP_035665256.1 | 385 |  |  |  | 88-356 | 1-41 |  |  |
|  |  |  | LOC118406448 | XP_035662392.1 | 1250 |  | 55-74;78-101;150-169;173-196;245-264;268-287;291-315 |  | 956-1222 |  |  | **ZU5**: 448-546 |
|  |  |  | LOC118408279 | XP_035664857.1 | 678 |  | 95-111; 115-134; 138-158; 161-180; 184-203; 256-275; 279-298; 302-321 |  | 392-656 |  |  |  |
|  |  |  | LOC118408570 | XP_035665271.1 | 593 |  | 14-37; 63-86; 109-132 |  | 307-568 | 164-259 |  |  |
|  |  |  | LOC118408558 | XP_035665259.1 | 918 |  | 52-68; 72-91; 95-114; 118-137; 141-160; 164-183; 187-206; 210-233; 258-277; 281-300; 304-323 |  | 633-894 | 495-586 |  |  |
|  |  |  | LOC118408552 | XP_035665254.1 | 801 |  | 38-54; 58-77; 81-100; 104-123; 127-146; 150-169; 173-192; 196-215; 219-238; 242-265; 291-310; 314-336; 337-356 |  | 535-796 | 392-487 |  |  |
|  |  |  | LOC118408550 | XP_035665252.1 | 1210 |  | 38-54; 58-77; 81-100; 104-123; 127-146; 150-169; 166-188; 189-208; 212-231; 235-254; 304-323; 327-346; 350-369; 375-398; 873-889; 893-912; 916-935; 939-958; 962-981; 985-1004; 1008-1027; 1031-1050; 1054-1073; 1100-1119; 1123-1142; 1146-1165; 1171-1192 |  | 548-809 | 405-500 |  |  |
|  |  |  | LOC118408562 | XP_035665264.1 | 758 |  | 37-53; 57-76; 80-99; 103-125; 126-145; 152-171; 224-243; 247-269; 270-289 |  | 468-729 | 325-420 |  |  |
|  |  |  | LOC118408547 | XP_035665248.1 | 1373 |  | 38-54; 58-77; 81-100; 104-123; 127-146; 150-173; 186-205; 209-228; 232-251; 255-274; 278-297; 301-324; 350-369; 386-415; 894-913 |  | 592-653; 1083-1344 | 451-546; 949-1044 |  |  |
|  |  |  | LOC118408560 | XP_035665262.1 | 891 |  | 38-54;58-77;82-104;104-123; 127-146; 150-169; 173-192; 196-215; 219-238; 242-261; 265-284; 288-311; 357-376; 403-422 |  | 601-862 | 458-553 |  |  |
|  |  |  | LOC118408566 | XP_035665268.1 | 845 |  | 89-112;115-131;135-154;158-177; 181-204; 216-235; 239-258; 262-285; 311-330; 334-353; 357-376 |  | 555-816 | 412-507 |  | **SH3**: 7-62 |
|  |  |  | LOC118409909 | XP_035667179.1 | 2143 | 270-299; 302-331; 335-364; 367-397; 399-428 | 510-529; 563-582; 604-623; 651-667; 696-715; 720-741; 792-811; | 877-1308 | 1494-1810 |  |  |  |
|  |  |  | LOC118416238 | XP_035677216.1 | 2597 | 678-707;710-740 | 1187-1210; 1211-1234; 1258-1277; 1282-1305; 1306-1325; 1330-1349; 1354-1382 | 1432-1835 | 1972-2221 |  |  | **ARM**: 130-172; 173-216; 459-501 |
|  |  |  | LOC118422684 | XP_035686272.1 | 2680 | 43-72; 79-108; 112-141; 180-211; 213-242; 248-277; 296-325; 355-384; 409-439 | 563-582; 587-606; 614-633; 637-656; 660-679; 788-811; 812-930; 987-1006 | 1113-1495 | 1972-2266 |  |  |  |
| Arthropoda | *D. grimshawi* | Hawaiian fruitfly |  | XP_032596557.1 | 2469 | 81-112; 114-143; 148-178; 310-339; 359-389 | 495-518; 518-537; 544-563; 567-586;590-609; 684-703; 709-731; 731-750; 805-828; 852-873; 875-899; | 992-1423 | 1748-2045 |  |  |  |
| Nematomorpha | *C. elegans* | Worm | Irk-1 | NP_492839.4 | 2393 | 56-86; 90-119; 197-226; 230-259; 264-293; 317-347; 361-390; 407-437 | 530-553; 579-601; 602-625; 626-650; 740-763; 854-878; 881-904 | 977-1428 | 1738-1986 |  |  |  |
| Cnidaria | *H. vulgaris* | Fresh-water polyp |  | XP_012555867.1 | 2064 | 54-84; 88-117; 133-164; 185-214; 225-254 | 340-362; 412-435;504-528; 583-605; | 662-1096 | 1244-1541 |  |  |  |
|  |  |  |  | XP_012555367.1 | 1746 | 4-36; 41-70; 74-103; 162-192 | 340-362; 363-384; 387-409; 431-455 | 492-931 | 1072-1339 |  |  |  |
|  |  |  |  | XP_012560904.1 | 2121 | 353-382; 385-415 | 602-625; 626-649; 719-742; 743-769; 795-818; 868-892 | 937-1350 | 1479-1735 |  |  |  |
|  |  |  |  | XP_012557001.1 | 1643 |  | 335-358; 359-381; 387-410; 429-453 | 481-870 | 1004-1258 |  |  |  |
| Amoebozoa | *D. discoideum* | Soil-dwelling amoeba | pats1 | XP_645923.1 | 3184 |  | 1389-1413; 1414-1436; 1437-1460; 1465-1487; 1490-1512; 1539-1561; 1562-1584; 1585-1606; 1608-1630; 1631-1654; 1678-1701 | 1710-2127 | 2247-2511 |  | 2780-2820; 2900-2937; 2939-2977; 2980-3031 |  |
| Cryptophyta | *G. theta* | Cryptomonad algae |  | XP_005818488.1 | 302 |  |  |  | 44-299 |  |  |  |
|  |  |  |  | XP_005834159.1 | 682 |  |  |  | 179-432 |  |  | **fh3**: 5-56; **FN3**: 67-155 |
| Fungi |  |  |  | N |  |  |  |  |  |  |  |  |
| Plants | *R. argentea* | Silver myrtle | Lrrk1-like | XP_030537727.1 | 2699 | 353-382;385-415 | 602-625; 626-649; 719-742; 743-769; 795-818; 868-892 | 937-1350 | 1479-1735 |  |  |  |

N means not found
